# Global Occurrence, Co-Contamination Patterns, and Toxic Effects of Mycotoxins in Fish Feed: A Multi-Species Perspective Review

**DOI:** 10.3390/toxins18090383

**Published:** 2026-09-06

**Authors:** Queenta Ngum Nji, Patrick Berka Njobeh

**Affiliations:** Department of Biotechnology and Food Technology, University of Johannesburg, Doornfontein Campus, Johannesburg 2094, South Africa; pnjobeh@uj.ac.za

**Keywords:** aquaculture, fish feed, fungal contamination, mycotoxin, toxicity, food safety

## Abstract

Aquaculture has become the fastest-growing food-production sector worldwide, with increasing reliance on plant-based feed ingredients such as maize, soybean, wheat, rice, and oilseed meals. Although these ingredients reduce dependence on fishmeal, they are highly susceptible to fungal contamination and subsequent mycotoxin production. Global surveys reveal widespread contamination of aquafeeds with major regulated mycotoxins, including aflatoxins, fumonisins, deoxynivalenol, zearalenone, ochratoxin A, and T-2 toxin, alongside emerging toxins such as enniatins, beauvericins, and sterigmatocystin metabolites. Aflatoxin B_1_ is the most prevalent and toxic mycotoxin, with contamination prevalence often exceeding 80–100% in Africa and Asia with contamination levels surpassing 400 µg/kg in some regions of these continents. Fumonisins, deoxynivalenol, and zearalenone are also commonly detected, with fumonisins levels occasionally exceeding 7000 µg/kg. Mycotoxins induce diverse toxic effects in fish, including growth suppression, hepatotoxicity, nephrotoxicity, immunosuppression, oxidative stress, reproductive dysfunction, and mortality. Collectively, these findings highlight the urgent need for improved surveillance, feed management, and harmonized regulations addressing multi-mycotoxin exposure to safeguard aquaculture productivity, fish, and public health. This review synthesizes current knowledge on the global mycotoxin contamination patterns, analytical detection methods in fish feed and processed seafood as well as the toxicological effects of mycotoxins, with emphasis on species-specific responses and food safety implications.

## 1. Introduction

Global aquaculture has expanded rapidly over recent decades and now represents one of the fastest-growing food production sectors worldwide. By 2022, global aquaculture production reached 185.4 million tonnes, with fish farming contributing over 53.4 million tonnes, led mainly by Asia, followed by the Americas, Africa, Europe, and Oceania [1]. Earlier reports indicated that aquaculture already supplied more than half of global seafood consumption and contributed approximately 20% of animal protein intake for over 3.3 billion people worldwide [2,3]. This growth has been supported by genetic improvement, water quality management, reproductive technologies, and feed formulation strategies [4,5,6]. Fish and other seafoods are recognized as important sources of high-quality protein, omega-3 fatty acids, vitamins, and essential minerals, making aquaculture critical for global food security [7].

The increasing demand for aquaculture products has intensified the need for nutritionally balanced formulated feeds, which account for 40–80% of production costs under intensive systems [8,9]. Factually, fishmeal was the dominant protein source in aquafeeds; however, the rapid expansion of aquaculture, increasing global demand for animal protein associated with population growth, sustainability concerns regarding marine resources, and the rising cost and limited availability of fishmeal have collectively driven the replacement of animal-derived ingredients with plant-based materials such as maize, soybean meal, wheat, rice, sorghum, and oilseed cakes [10,11]. Although these ingredients provide affordable protein and energy, they are highly susceptible to fungal invasion and subsequent mycotoxin accumulation during cultivation, harvesting, storage, and transportation. Consequently, the increasing incorporation of plant-based ingredients in aquafeeds may elevate the risk of fungal growth and mycotoxin contamination because these materials are often more susceptible to fungal colonization during cultivation, harvesting, processing, transport, and storage [12,13]. This risk can be further exacerbated by climate-related factors such as increased temperature and humidity, poor storage conditions, inappropriate handling practices, and inadequate hygiene during feed production and storage. Mycotoxins are toxic secondary metabolites produced primarily by fungi belonging to the genera *Aspergillus*, *Fusarium*, *Claviceps*, and *Penicillium* [14]. These fungi commonly contaminate cereals and oilseed crops used in aquafeeds and produce a wide range of toxins, including aflatoxins (AFs), fumonisins (FBs), ochratoxin A (OTA), zearalenone (ZEA), trichothecenes such as deoxynivalenol (DON), enniatins (ENs), and ergot alkaloids [15]. Mycotoxin contamination is influenced by environmental factors such as temperature, humidity, moisture content, insect damage, and storage conditions [16,17,18,19]. Tropical and subtropical climates are particularly favorable for fungal growth, especially under temperatures above 27 °C, humidity exceeding 62%, and feed moisture levels above 14% [20]. Importantly, mycotoxins are highly thermostable compounds that often survive feed heating and other food processing methods, thereby persisting throughout the food chain [21].

Fish comprise a taxonomically and ecologically diverse group with distinct feeding strategies and dietary protein requirements. Freshwater omnivorous species, including common carp (*Cyprinus carpio*), Nile tilapia (*Oreochromis niloticus*), rainbow trout (*Oncorhynchus mykiss*), and African catfish (*Clarias gariepinus*), are typically fed diets in which plant-derived ingredients (maize, soybean meal, wheat, rice bran, cottonseed meal, and sunflower meal) constitute approximately 50–90% of the formulation [22,23]. These plant-derived feed ingredients are susceptible to fungal colonization because they contain readily available carbohydrates and lipids and may be exposed to elevated moisture, temperature fluctuations, insect damage, mechanical injury, and prolonged storage during cultivation, harvesting, transport, processing, and storage. Under favorable conditions, toxigenic fungi including *Aspergillus*, *Fusarium*, and *Penicillium* species can proliferate and produce AFs, FBs, DON, ZEAs, OTA, and other mycotoxins [24,25]. Non-toxigenic fungi may also colonize these ingredients and are not necessarily associated with mycotoxin production; however, the present review focuses specifically on colonization by mycotoxin-producing fungi because such colonization represents the primary toxicological concern for aquafeed safety. In contrast, marine carnivorous species such as Atlantic salmon (*Salmo salar*) and sea bass (*Dicentrarchus labrax*), have historically relied on fishmeal and fish oil-based diets, which were associated with a relatively lower risk of contamination by plant-associated mycotoxins [10,23]. However, the increasing demand for sustainable aquaculture and rising cost of marine-derived feed ingredients (nutritious products harvested from marine organisms like fish, krill, shellfish, and algae), has accelerated the replacement of fishmeal and fish oil with plant proteins and vegetable oils, thereby increasing the likelihood of mycotoxin contamination even in feeds formulated for carnivorous marine species [10,24]. Consequently, the occurrence, concentration, and co-occurrence of mycotoxins in aquafeeds cannot be generalized across all fish species but are largely influenced by species-specific dietary formulations, ingredient sourcing, geographic location, climatic conditions, storage practices, and feed manufacturing processes [25,26]. Therefore, interpreting mycotoxin occurrence and associated toxicological risks in aquaculture requires consideration of species-specific feeding ecology and production systems rather than treating fish as a homogeneous group.

Recognition of mycotoxins as a major feed safety concern evolved progressively with the intensification of aquaculture and increasing use of plant ingredients. Early global estimates suggested that approximately 25% of crops worldwide were contaminated annually by mycotoxins; however, more recent studies indicate contamination rates may exceed 85% in feed raw materials and compounded feeds [27]. Surveys conducted across continents consistently demonstrate widespread contamination of aquafeeds, with multiple mycotoxins occurring simultaneously [28,29]. Regional contamination patterns are often linked to climatic conditions and feed production systems, with similarities observed between Asia and the Americas, whereas Africa and Europe exhibit distinct contamination profiles. However, data from Sub-Saharan Africa, Australia, and others remain relatively scarce despite rapid aquaculture expansion in the region [16,30,31].

Growing awareness of feed contamination has revealed the widespread global ocurrence of mycotoxins particularly AFB_1_, OTA, and ZEA in aquaculture feeds and processed fish products, highlighting significant food safety concerns for both aquaculture and human consumption. In parallel, research attention has shifted toward understanding the biological and toxicological consequences of mycotoxin exposure in fish species. Experimental and field studies have shown that AFs impair growth performance, reduce feed utilization efficiency, suppress immune responses, damage vital organs, and increase mortality in fish [32]. Aflatoxins, particularly AFB_1_, are strongly hepatotoxic and carcinogenic, causing liver lesions, oxidative stress, and reduced growth. OTA is associated with nephrotoxicity and immunosuppression, whereas ZEA exhibits estrogenic and reproductive effects. Trichothecenes and FBs interfere with protein synthesis, intestinal integrity, and metabolic functions although the severity of these effects varies among fish species and exposure conditions [24,33]. Toxicity severity varies considerably depending on fish species, age, exposure duration, feeding behavior, and environmental conditions, with the most severe effects including hepatotoxicity, nephrotoxicity, oxidative stress, immunosuppression, impaired growth and feed efficiency, reproductive dysfunction, histopathological damage to liver and kidney tissues, and increased mortality, particularly during chronic exposure or combined exposure to multiple mycotoxins [34]. Controlled toxicological studies indicate that co-occurrence of multiple mycotoxins may result in additive or synergistic toxic effects that exceed predictions based on single-toxin exposure [35]. Concerns regarding food safety further intensified when studies demonstrated that certain mycotoxins and their metabolites can accumulate in edible fish tissues. Experimental investigations detected AFB_1_ residues in fish liver and muscle tissues [36,37]. Because fish is an important dietary protein source worldwide, the transfer of mycotoxins from contaminated aquafeeds to fish edible tissues warrants attention from a food-safety perspective. Experimental studies have shown that AFs, OTA, and certain *Fusarium* toxins can be detected in liver, kidney, muscle, or other tissues of exposed fish, although carryover is generally low and varies with mycotoxin type, fish species, dietary concentration, exposure duration, and metabolic detoxification capacity. Chronic exposure may therefore contribute to consumer dietary intake, particularly in populations with high fish consumption, supporting the need for monitoring mycotoxins in both aquafeeds and aquaculture products.

Given the increasing global dependence of aquaculture on plant-based feed ingredients and the widespread occurrence of fungal contamination, a comprehensive understanding of mycotoxin risks in fish feed and seafood products has become urgently necessary. Although previous studies have investigated different aspects of mycotoxin contamination, most have focused on isolated components such as occurrence, toxicology, or detection methods. In contrast, this review provides an integrated and up-to-date framework that combines global contamination occurrence, co-contamination patterns, fungal ecology, emerging mycotoxins, analytical detection techniques, species-specific toxicological responses, and public health implications. By integrating contamination data with toxicological outcomes across multiple fish species and production systems, this review seeks to provide a more realistic understanding of multi-mycotoxin exposure under practical aquaculture conditions. Furthermore, this review aims to support risk assessment, improve feed safety management and surveillance strategies, identify critical knowledge gaps, and contribute to the development of evidence-based and harmonized regulatory frameworks for managing multi-mycotoxin exposure in aquaculture systems, thereby supporting sustainable aquaculture production, seafood safety, and public health protection.

## 2. Common Types of Fungi Isolated from Fish Feed

Fungal contamination of fish feed is a widespread and persistent challenge in aquaculture because the fungal communities colonizing feed ingredients largely determine the diversity and abundance of mycotoxins subsequently detected in finished feeds. Unlike bacteria, many filamentous fungi not only deteriorate feed quality through nutrient degradation but also synthesize toxic secondary metabolites that remain stable during feed processing and storage [27,38]. Consequently, understanding the fungal ecology of aquafeeds provides the biological basis for interpreting the occurrence and distribution of mycotoxins discussed in Section 4.

Across most geographical regions included in this review, *Aspergillus* species were consistently the dominant fungal contaminants of fish feed (Supplementary Table S1). Among these, *Aspergillus flavus* was the most frequently isolated species, with high prevalence reported in Egypt (48–56%) [39,40], Iran (47%) [32], and Brazil (35–61%) [41,42]. The predominance of *A. flavus* is of particular significance because it is the principal producer of AFs, especially AFB_1_, which is considered the most toxic AF in aquaculture. Adverse effects such as reduced growth, impaired feed efficiency, liver lesions, oxidative stress, and increased mortality have been reported in fish fed diets containing approximately 20–1000 µg AFB_1_/kg feed, with chronic effects frequently observed at concentrations below 100 µg/kg [24,38]. In addition to its high toxicity, AFB_1_ is economically important because contamination of aquafeeds can reduce productivity, increase health-management costs, and cause rejection of contaminated feed ingredients and aquaculture products. Other frequently isolated species included *A. parasiticus*, another major producer of AFs, *A. ochraceus* and *A. carbonarius*, which produce OTA, and *A. versicolor*, a recognized producer of sterigmatocystin [38,43]. Although *A. niger*, *A. fumigatus*, *A. terreus*, and *A. nomius* differ in their mycotoxin-producing capacity, their occurrence indicates the presence of a fungal community capable of producing multiple secondary metabolites under favourable environmental conditions.

The predominance of *Aspergillus* species in fish feeds reflects their ecological adaptation to storage environments rather than field conditions. Commercial aquafeeds generally contain low moisture levels (typically <12%) but are rich in carbohydrates, proteins, and lipids derived from cereals and oilseed meals. These conditions favour xerophilic and thermotolerant fungi such as *Aspergillus*, which can colonize substrates at relatively low water activity and temperatures commonly encountered during storage and transportation [38]. The rapid expansion of aquaculture, driven by increasing global demand for animal protein associated with population growth, sustainability concerns regarding marine resources, and the rising cost and limited availability of fishmeal, has promoted the replacement of animal-derived ingredients with plant-based materials such as maize, soybean meal, wheat, rice, sorghum, peanut meal, cottonseed meal, and rice bran. This increasing use of plant-derived ingredients has consequently expanded the availability of substrates susceptible to colonization by *Aspergillus* species which also favours other fungal growth [10,24]. Consequently, the widespread occurrence of AFs reported in aquafeeds globally is consistent with the predominance of *Aspergillus* observed in fungal isolation studies.

The second most frequently isolated fungal genus was *Penicillium*, with high isolation frequencies reported in Brazil (83%) [44], Iran (42%) [32], and Egypt (22–40%) [39,40]. Species including *P. citrinum*, *P. expansum*, *P. crustosum*, and *P. chrysogenum* were commonly identified. These fungi are important because they produce several mycotoxins, including citrinin (CIT), patulin (PAT), roquefortine C, and penitrem A, while some species also contribute to OTA production [43]. Similar to *Aspergillus*, *Penicillium* species can colonize stored feed ingredients when moisture and water activity are not adequately controlled, particularly during prolonged storage, highlighting the importance of proper drying and storage practices [38]. Their frequent co-occurrence with *Aspergillus* suggests that finished aquafeeds may contain complex mixtures of storage-associated mycotoxins.

In contrast, *Fusarium* species represent typical field fungi that infect cereal crops before harvest and subsequently enter the aquafeed production chain through contaminated plant ingredients. *Fusarium* spp. were frequently isolated in Iran (26%), Portugal (25%), and Mexico [32,45,46]. Unlike many storage-associated fungi, several *Fusarium* species are primarily associated with crop production and can contaminate plant-derived ingredients before harvest or during subsequent handling. Species within this genus are capable of producing a diverse range of mycotoxins, including FBs, DON, ZEA, and T-2 toxins [27]. Their presence in fish feeds therefore reflects contamination originating from agricultural production rather than feed storage. As the inclusion of maize and wheat in commercial aquafeeds has increased over recent decades, contamination by *Fusarium* mycotoxins has become increasingly common, particularly in temperate regions where these fungi dominate cereal production systems [47].

Other fungal genera, including *Eurotium*, *Cladosporium*, *Mucor*, *Rhizopus*, *Alternaria*, *Phoma*, *Nigrospora*, *Aureobasidium*, and yeasts such as *Candida*, were also identified across multiple studies (Supplementary Table S1). Although many of these genera are not primary producers of toxigenic mycotoxins in aquafeeds, their occurrence provides valuable indicators of feed hygiene, storage quality, and environmental contamination. For example, xerophilic *Aspergillus* species (formerly classified in the genus *Eurotium*) are highly adapted to low-water-activity environments and therefore indicates prolonged storage under dry conditions, whereas *Cladosporium* and *Alternaria* species may introduce emerging mycotoxins such as alternariol and tenuazonic acid into feed ingredients [28,43].

The composition of fungal communities varied geographically, reflecting differences in temperature, relative humidity, moisture availability (water activity), feed ingredients, and storage practices. Studies from warm and humid regions in North Africa, the Middle East, South America, sub-Saharan Africa, and Southeast Asia frequently reported a predominance of *Aspergillus* species, particularly under storage conditions characterized by temperatures above 25–30 °C and relative humidity exceeding approximately 70%, which favour fungal growth and AF production [48]. For example, high frequencies of *Aspergillus* species have been reported in feed and feed ingredients from Egypt, Brazil, Iran, and several other tropical and subtropical regions. In contrast, surveys from temperate cereal-producing regions, including Argentina, southern Brazil, Uruguay, and parts of Europe such as Italy, Serbia, Hungary, and Croatia, more often detected *Fusarium*, *xerophilic Aspergillus* species (formerly classified as *Eurotium*), and *Cladosporium*. These patterns are consistent with cooler growing seasons, higher cereal production, and storage environments with lower temperatures and reduced moisture availability [47]. Feed formulation also influenced fungal diversity, with tilapia feeds containing high proportions of maize and oilseed meals frequently dominated by *A. flavus* and *Penicillium* spp., whereas rainbow trout feeds exhibited more diverse fungal communities reflecting differences in ingredient composition and production systems.

## 3. Composition and Characteristics of Aquafeeds and Their Implications for Mycotoxin Contamination

The susceptibility of aquafeeds to mycotoxin contamination is largely determined by their ingredient composition, manufacturing processes, and storage conditions. Fish feeds were formulated primarily from marine-derived ingredients such as fishmeal and fish oil, which provided high-quality protein and lipid sources with relatively low susceptibility to contamination by plant-associated mycotoxins [10,23]. However, increasing pressure on wild fisheries, rising costs of marine ingredients, and the need to improve the sustainability of aquaculture have driven a global transition toward plant-based feed formulations [49]. Therefore, commercial aquafeeds contain substantial proportions of cereal grains and oilseed meals, including maize, wheat, soybean meal, cottonseed meal, sunflower meal, rapeseed meal, rice bran, peanut meal, and various cereal by-products, all of which are recognized substrates for toxigenic fungi belonging primarily to the genera *Aspergillus*, *Fusarium*, and *Penicillium* [24,27]. Hence, aquafeeds have become increasingly vulnerable to contamination by both regulated (AFs, FBs, DON, ZEA, T-2 toxins, OTA) and emerging mycotoxins (ENs, STC, MON).

Aquafeeds are commonly classified as starter, grower, and finisher feeds according to the developmental stage of the cultured fish, and each category is formulated to meet the specific nutritional requirements associated with that stage as well as the production system and feeding guild of the species, including carnivorous, omnivorous, and herbivorous fish [22]. Carnivorous species such as Atlantic salmon (*Salmo salar*) and rainbow trout (*Oncorhynchus mykiss*) often require diets containing approximately 40–50% crude protein and 15–30% lipid, whereas omnivorous species such as Nile tilapia (*Oreochromis niloticus*), common carp (*Cyprinus carpio*), and African catfish (*Clarias gariepinus*) are generally fed diets containing approximately 25–40% crude protein and 5–15% lipid and can utilize greater proportions of plant-derived ingredients [22,23]. Nevertheless, the gradual increase in the inclusion of plant-derived protein ingredients and the corresponding reduction in fishmeal use in aquafeeds over recent decades has diminished the traditional differences between marine and freshwater feed formulations [10,50]. For example, fishmeal inclusion in salmonid feeds has declined from more than 40–50% in the 1990s to often less than 15–20% in many modern commercial diets, with soybean meal, maize gluten meal, wheat products, and other cereal-based ingredients increasingly used as substitutes [10,50]. This shift has increased the potential exposure of both marine and freshwater species to cereal-associated mycotoxins such as AFs, FBs, DON, and ZEA.

Compared with feeds for terrestrial livestock, aquafeeds differ in ingredient composition, nutrient density, lipid sources, and the aquatic environment, all of which can influence both mycotoxin occurrence and toxicological outcomes. Poultry and swine diets are typically formulated largely from cereal grains and oilseed meals, making contamination by *Fusarium*- and *Aspergillus*-derived mycotoxins a long-recognized challenge [28,51]. In contrast, many aquafeeds have shifted from formulations rich in marine ingredients, such as fishmeal and fish oil, to diets containing higher proportions of plant-derived ingredients, including soybean meal, maize products, wheat products, and other cereal- and oilseed-based materials. This transition has occurred progressively since the early 2000s, as fishmeal inclusion levels declined and plant-protein use increased in commercial aquaculture feeds. Consequently, the mycotoxin exposure profile of aquaculture has changed, with increasing reports of AFs, FBs, DON, ZEA, OTA, and emerging mycotoxins in commercial fish feeds [24,25]. Aquatic production systems are also characterized by continuous exposure to waterborne environmental conditions, and stressors such as poor water quality, temperature fluctuations, hypoxia, and infectious agents may interact with dietary mycotoxins and exacerbate oxidative stress, immune dysfunction, and growth impairment [25,52]. Similar stressor–toxin interactions can occur in terrestrial livestock, but the nature and intensity of environmental exposure differ between aquatic and terrestrial production systems. In addition, several fish species exhibit lower hepatic biotransformation capacity for certain mycotoxins than poultry. For example, rainbow trout (*Oncorhynchus mykiss*) are highly sensitive to AFB_1_ because of limited detoxification through glutathione conjugation and efficient formation of the reactive AFB_1_-8,9-epoxide, whereas poultry generally show greater metabolic detoxification capacity for this toxin. As a result, chronic dietary exposure to relatively low aflatoxin concentrations can produce significant toxic effects in susceptible fish species [24].

The occurrence of mycotoxins in aquafeeds is also influenced by the complexity of feed supply chains. Commercial fish feeds commonly contain ingredients sourced from multiple countries and climatic regions, allowing raw materials with distinct fungal communities and contamination profiles to be blended during manufacture [27]. Consequently, aquafeeds frequently contain multiple mycotoxins simultaneously, including both regulated (mycotoxins which are routinely monitored with set limits) toxins such as AFB_1_, FBs, DON, ZEA, and OTA, together with emerging (mycotoxins not routinely monitored) metabolites including ENs, BEA, MON, and Alternaria toxins [28,29].

Reports further demonstrate that mycotoxin contamination in aquafeeds has evolved alongside changes in feed formulation and analytical technologies. Earlier investigations conducted during the 1980s and 1990s predominantly focused on AFs because these were the most recognized contaminants and analytical methods such as thin-layer chromatography (TLC), enzyme-linked immunosorbent assays (ELISA), and high-performance liquid chromatography (HPLC) had easily available simplified protocols and standards tailored towards AFs [24]. Since the widespread adoption of liquid chromatography coupled with tandem mass spectrometry (LC-MS/MS), comprehensive multi-mycotoxin analyses have become possible, revealing that commercial aquafeeds routinely contain complex mixtures of regulated, modified, and emerging mycotoxins rather than single contaminants [27,28]. Concurrently, the increasing incorporation of plant-derived feed ingredients has contributed to higher frequencies of contamination by *Fusarium* toxins, while climate change and globalization of feed ingredient trade have further diversified regional contamination patterns [53,54]. These developments indicate that mycotoxin contamination in aquafeeds has shifted from an isolated AF problem to a complex multi-mycotoxin challenge requiring integrated surveillance, improved analytical methods, and species-specific risk assessment.

## 4. Occurrence, Distribution, and Detection of Mycotoxins in Fish Feed

Geographic location and climate are major determinants of mycotoxin contamination in aquafeeds because they influence fungal ecology, mycotoxin biosynthesis, crop production systems, and post-harvest storage conditions [47,48]. Globally, aquaculture production and aquafeed manufacturing are concentrated in Asia, which accounted for approximately 91% of world aquaculture output in 2022 [1]. Within Asia, China contributed about 57%, India 8%, Indonesia 7%, Vietnam 5%, Bangladesh 3%, and Thailand about 1% of global production, together representing more than 80% of global aquaculture output and the largest market for plant-derived feed ingredients [1]. Europe contributed approximately 3% of global aquaculture production, led by Norway (1.5%), Spain (0.3%), and Greece (0.2%) [1]. The Americas contributed roughly 5%, including Chile (~1.1%), Brazil (~0.7%), the United States (0.3%), and Canada (0.1%) [1]. Africa contributed approximately 3%, with Egypt (1.6%) accounting for most of the continent’s production, followed by Nigeria, Uganda, Ghana, and South Africa [1]. Oceania contributed less than 1%, with Australia being the principal producer in the region [1]. Across these regions, the increasing replacement of fishmeal and fish oil with plant-derived ingredients including maize, wheat, soybean meal, cottonseed meal, sunflower meal, rice bran, and peanut meal has substantially increased the susceptibility of aquafeeds to contamination by fungal secondary metabolites [10,24]. The occurrence and composition of mycotoxins in aquafeeds are strongly influenced by regional climatic conditions. Tropical and subtropical climates, characterized by high temperatures (25–35 °C), elevated relative humidity (>70%), and seasonal rainfall, favour the growth of *Aspergillus* species and are therefore predominantly associated with AFs, particularly AFB_1_, as well as ochratoxin A (OTA) [27,48]. Such environmental conditions are common in many aquaculture-producing countries across Southeast Asia, sub-Saharan Africa, and parts of South America, where inadequate drying and poor storage practices further increase the risk of fungal proliferation and mycotoxin accumulation [24,55]. In contrast, temperate regions of Europe and North America generally experience cooler growing seasons and moderate rainfall that favour *Fusarium* species, resulting in higher contamination of cereal-based feed ingredients with fumonisins (FBs), deoxynivalenol (DON), zearalenone (ZEA), T-2 toxin, and HT-2 toxin [27,47]. Consequently, the mycotoxin profile of aquafeeds often reflects the climatic conditions and dominant fungal communities in the regions where feed ingredients are cultivated rather than the location where the feeds are manufactured.

Climate change is expected to further reshape the global distribution of mycotoxigenic fungi through increasing temperatures, altered precipitation patterns, prolonged droughts, and more frequent extreme weather events, thereby influencing both the occurrence and co-occurrence of mycotoxins in aquaculture feed ingredients [53,54]. For example, severe drought followed by periods of high humidity has repeatedly been associated with elevated AF contamination in maize, with outbreaks in several African countries reporting concentrations that exceeded regulatory limits by several-fold during drought years [53,54]. In Europe, climate-based models predict that warming scenarios of +2–5 °C could increase the suitability of southern and parts of central Europe for *Aspergillus flavus*, potentially shifting AF risk northward into areas that historically experienced predominantly *Fusarium*-associated contamination [56,57]. Experimental studies have further shown that elevated temperature (30–35 °C) combined with water stress can significantly increase aflatoxin B_1_ production by *A. flavus* compared with cooler, non-stressed conditions [53,57]. Globalization of feed ingredient supply chains also contributes to multi-mycotoxin exposure. The aquafeed industry increasingly relies on internationally traded commodities such as maize, soybean meal, wheat, rice bran, and oilseed meals sourced from multiple climatic regions. According to FAO trade statistics, hundreds of millions of tonnes of cereals and oilseeds are traded internationally each year, creating opportunities for ingredients with different fungal and mycotoxin profiles to be blended during feed manufacture [1]. Surveys of commercial aquafeeds frequently report the simultaneous presence of several regulated and emerging mycotoxins, with co-occurrence detected in more than 50–80% of analyzed samples in some studies [27,28]. Consequently, climate-driven changes in crop contamination combined with increasingly globalized feed supply chains are likely to increase the complexity and variability of mycotoxin contamination patterns in aquaculture feeds.

Consequently, mycotoxin contamination in aquafeeds has become a global challenge with significant implications for fish health, aquaculture productivity, and food safety. The compiled dataset in the present review demonstrates that mycotoxin contamination occurs across diverse geographical regions, aquaculture production systems, fish species, and feed formulations, with considerable variability in contamination frequencies, concentration ranges, and analytical detection methods (Table 1). Large-scale surveillance studies further indicate that contamination rarely involves a single mycotoxin but is frequently characterized by the co-occurrence of multiple regulated and emerging toxins. For instance, a comprehensive global survey by Gruber-Dorninger, Müller Gruber-Dorninger, Müller [29] reported high prevalence rates for AFB_1_ (41.6%), FBs (68.6%), DON (58.0%), ZEA (61.1%), and OTA (33.6%), together with emerging mycotoxins such as ENs (46.9–54.0%) and BEA (35.0%) (Table 1). These findings underscore the complexity of mycotoxin contamination in aquafeeds, where fish are commonly exposed to mixtures of regulated and emerging mycotoxins that may exert additive or synergistic toxic effects, emphasizing the need for region-specific surveillance, risk assessment, and mitigation strategies.

Early investigations primarily focused on regulated mycotoxins such as AFs, FBs, DON, ZEA, OTA, etc.; however, advances in analytical technologies have since revealed the widespread presence of emerging mycotoxins, including ENs, BEA, *Alternaria* toxins, moniliformin (MON), and sterigmatocystin (STC). Global surveys have shown AF contamination in approximately 41.6% of analyzed fish feed samples [29], although regional studies often report substantially higher prevalence rates. In several African and Asian countries, contamination levels reached 95–100% of analyzed samples [62,69,76,80] and mycotoxin concentrations varied considerably depending on climatic conditions, storage practices, and feed composition. In Egypt, relatively lower AFB_1_ concentrations ranging from 0.07 to 20 µg/kg were reported in commercial aquafeed ingredients [40], whereas East African studies documented AFB_1_ concentrations ranging from 97 to 403.4 µg/kg [58]. Kenya reported an AF prevalence of 83.95%, with concentrations ranging from 0.07 to 39.7 µg/kg [61], while Iran recorded AFB_1_ levels up to 68.5 µg/kg [32]. In Europe, surveys of commercial aquafeeds and feed ingredients originating mainly from Norway, Spain, Italy, Greece, and other Mediterranean countries generally reported lower AF prevalence (typically <30%) and concentrations mostly below 5 µg/kg, with many samples containing non-detectable levels [27,47]. These lower levels are attributed to routine regulatory monitoring and enforcement of maximum limits for AFs in feed, as well as improved feed management practices such as rapid drying after harvest, moisture control during storage, hygienic handling, ingredient traceability, and quality assurance programs [27,47]. The predominance of AFs in tropical and subtropical regions remains strongly associated with warm, humid conditions that favor *Aspergillus* growth, together with less effective monitoring and storage management in some production systems.

Following AFs, *Fusarium*-derived mycotoxins constitute another major category of contaminants in aquaculture feeds from the articles reviewed. Fumonisins (FB_1_ and FB_2_) are among the most prevalent *Fusarium* toxins, with global occurrence estimated at approximately 68.6% [29]. Concentrations vary widely across studies, ranging from moderate levels to extremely high values exceeding 7000 µg/kg. Gonçalves, Schatzmayr Gonçalves, Schatzmayr [91] reported FB concentrations up to 7534 µg/kg, while Kenyan studies documented levels reaching 2076.6 µg/kg [60]. Similarly, East African studies identified FB contamination of up to 2834.6 µg/kg [62], emphasizing the importance of monitoring *Fusarium* contamination in warm and humid climates. South America has also emerged as a hotspot for FB contamination, with surveys reporting prevalence rates as high as 76% [67,68].

Trichothecenes, particularly DON and its derivatives, such as DON-3-glucoside and 15-acetyl-DON (15-ADON), are also widely distributed in fish feeds globally. DON occurrence has been reported in approximately 58% of analyzed feed samples worldwide [29]. Although some studies reported relatively low DON concentrations ranging from 0.25 to 2.5 µg/kg [73], other investigations documented substantially higher levels of up to 819.9 µg/kg [60]. In several European and multinational Asian studies, DON contamination reached 100% prevalence at concentrations of 10–396 µg/kg [35,72,75], demonstrating its widespread occurrence in cereal-based feed ingredients. Northern Asia has been identified as a region with particularly high DON prevalence, reportedly reaching 83% [67,68].

Zearalenone, another important *Fusarium* toxin, is also commonly detected in aquafeeds. Global surveys indicate occurrence rates of approximately 61.1% [29], with concentrations ranging from trace levels to 757.9 µg/kg [60]. Studies conducted in Europe and Asia consistently reported high prevalence rates, with some surveys documenting occurrence of up to 63% [67,68]. The detection of ZEA metabolites, such as α-zearalenol and β-zearalenol, indicates that ZEA has undergone fungal biotransformation during crop growth or storage and suggests the simultaneous presence of multiple related mycotoxins within aquafeed ingredients, supporting the occurrence of co-contamination in feed systems.

Ochratoxin A, although less frequently detected than AFs and *Fusarium* toxins in aquafeed, remains an important contaminant due to its nephrotoxic and immunosuppressive properties. Global occurrence rates are estimated at 33.6% [29]. Concentrations vary considerably between regions and studies, ranging from very low levels of 0.01–0.28 µg/kg in Italy [77] to extremely high concentrations of 501.7 µg/kg [25]. OTA contamination is often associated with poor storage conditions and prolonged moisture exposure during feed handling and processing.

In recent years, growing attention has been directed toward emerging and less-regulated mycotoxins. Enniatins (ENA, ENB, ENB1, and ENA1) have been increasingly detected in European and global studies, with occurrence rates reaching 54% globally and concentrations of up to 186.7 µg/kg [29,60]. Beauvericin (BEA) has also emerged as a significant contaminant, occurring in approximately 35% of samples worldwide and reaching concentrations as high as 841.8 µg/kg [60]. *Alternaria* toxins, including alternariol (AOH) and alternariol monomethyl ether (AME), have been detected at moderate prevalence rates (~40.7%), with concentrations up to 94.5 µg/kg. Other metabolites, such as MON and STC, occasionally occur at exceptionally high concentrations exceeding 2000–3000 µg/kg, likely reflecting localized contamination events in heavily colonized feed ingredients and favorable storage conditions that promote fungal growth and toxin accumulation, highlighting the complexity and diversity of contamination profiles in fish feeds.

A major characteristic of fish feed contamination is the frequent co-occurrence of multiple mycotoxins within the same sample. This pattern is expected because aquafeeds are typically formulated from several plant-derived ingredients, including maize, wheat, soybean meal, rice bran, and oilseed meals, each of which may harbor different fungal metabolites. Multi-residue analytical studies repeatedly demonstrated simultaneous contamination by AFs, FBs, T-2, ZEA, OTA, and emerging mycotoxins in complete pelleted aquafeeds and compound feed ingredients collected from aquaculture farms and feed manufacturers. For example, Mwihia, Lyche Mwihia, Lyche [60] identified more than 30 different mycotoxins in fish feeds from East Africa, Gonçalves, Do Cam Gonçalves, Do Cam [72] reported simultaneous contamination with AFs, DON, FBs, ZEA, and OTA across multiple Asian countries. Similarly, Modrá, Šišperová Modrá, Šišperová [82] documented 100% multi-mycotoxin contamination in feed samples from the Czech Republic. Such co-occurrence is particularly concerning because combined exposure may result in additive or synergistic toxic effects, potentially intensifying adverse impacts on fish growth, immunity, organ function, and survival.

Geographical analyses reveal distinct contamination patterns linked to climatic conditions, agricultural practices, and storage systems. Africa and Asia generally exhibit the highest AF and FB contamination due to warm and humid conditions that favor fungal growth [61,62] and is exacerbated by poor handling and storage conditions. East African studies frequently report severe contamination with AFs, FBs, and DON, while Cameroon and Egypt also demonstrate high AF prevalence [40,59]. In contrast, Europe is characterized by higher occurrence of *Fusarium* toxins, such as DON and ZEA, consistent with temperate climatic conditions [67,68]. European studies additionally report frequent detection of emerging mycotoxins, such as ENs and BEA [79,81,84]. Asian countries, including Sri Lanka, India, Indonesia, Myanmar, Taiwan, and Thailand, consistently report widespread contamination with AFs, DON, ZEA, FBs, and OTA [72,80]. In the Americas, Brazil generally reports lower AF occurrence, whereas Argentina demonstrates high prevalence of multi-mycotoxin contamination involving FBs and DON [26].

Although Table 1 demonstrates that mycotoxin contamination in aquafeeds is a global phenomenon, the reported prevalence and concentration ranges vary considerably among studies. Rather than reflecting inconsistent contamination patterns, this variability is primarily attributable to differences in analytical methodologies, geographical and climatic conditions, feed composition, sampling strategies, and storage practices. Collectively, these factors determine not only the occurrence and concentration of individual mycotoxins but also the diversity of toxin mixtures detected in commercial aquafeeds.

One of the major sources of inter-study variability is the analytical methodology employed. Earlier studies predominantly relied on enzyme-linked immunosorbent assays (ELISA), thin-layer chromatography (TLC), high-performance liquid chromatography (HPLC), or fluorescence-based methods, which were generally designed to detect one or a limited number of regulated mycotoxins, principally aflatoxins and OTA [39,40,65]. ELISA remains widely used in resource-limited settings because of its simplicity and cost-effectiveness [59]. In contrast, more recent investigations have increasingly adopted liquid chromatography coupled with tandem mass spectrometry (LC-MS/MS), enabling the simultaneous quantification of hundreds of regulated, modified, and emerging mycotoxins with substantially improved sensitivity and selectivity [56,62,75]. Consequently, studies using LC-MS/MS consistently report a greater diversity of contaminants than those employing conventional analytical techniques. For example, the multi-mycotoxin surveys conducted by Mwihia, Lyche Mwihia, Lyche [60] and Gruber-Dorninger, Müller Gruber-Dorninger, Müller [29] identified not only AFs, FBs, DON, ZEA, and OTA, but also numerous emerging mycotoxins, including ENs, BEA, MON, STC, and Alternaria toxins. Therefore, apparent differences among studies may reflect analytical capability rather than genuine differences in contamination.

Geographical and climatic conditions represent another major determinant of the observed heterogeneity. Tropical and subtropical regions, particularly Africa, Southeast Asia, and parts of South America, consistently reported higher prevalence of AFs, which corresponds with the predominance of *Aspergillus* species under warm temperatures and high relative humidity [27,48]. For example, several African studies reported aflatoxin contamination frequencies exceeding 95%, with some investigations detecting contamination in every sample analysed [58,62,63]. In contrast, studies conducted in Europe more frequently detected *Fusarium*-derived mycotoxins such as DON, fumonisins, ZEA, T-2 toxin, and HT-2 toxin, reflecting the cooler temperate conditions that favour *Fusarium* species during cereal production [35,75,83]. These regional differences indicate that mycotoxin profiles largely reflect the climatic conditions under which feed ingredients are produced rather than the location where finished feeds are manufactured.

Ecological and feed formulation factors further contribute to the wide variation observed across studies. Aquafeeds are increasingly incorporate maize, wheat, soybean meal, rice bran, cottonseed meal, peanut meal, and other plant-derived ingredients, each possessing distinct susceptibilities to fungal colonization and mycotoxin contamination [10,24]. Diets formulated for omnivorous freshwater species generally contain higher proportions of cereals and oilseed meals than traditional marine carnivorous feeds, increasing the likelihood of contamination by both *Aspergillus*- and *Fusarium*-derived mycotoxins. In addition, variation in ingredient sourcing, harvesting conditions, moisture content, storage duration, feed processing, and transportation can substantially influence fungal proliferation and toxin production before feeds reach aquaculture facilities [38,47]. Consequently, even feeds produced within the same country may exhibit markedly different contamination profiles depending on raw material origin and post-harvest management.

Differences in study design also explain part of the observed variability. Sample size ranged from fewer than 10 samples to more than 400, while studies differed in the type of feed analysed (commercial versus farm-formulated feeds), target fish species, production systems, sampling season, and sampling location. Smaller studies may overestimate or underestimate contamination because of limited representativeness, whereas large-scale surveillance programmes provide more reliable estimates of regional prevalence. Furthermore, some investigations reported only the presence of target mycotoxins, whereas others quantified concentration ranges or included modified and emerging metabolites, making direct comparison among studies challenging.

Despite these differences, several consistent patterns emerge from the compiled literature. First, AFs remain the most frequently investigated mycotoxins worldwide, reflecting both their toxicological importance and the widespread occurrence of *Aspergillus* species in aquafeeds. Second, multi-mycotoxin contamination is considerably more common than contamination by a single toxin, particularly in studies employing LC-MS/MS. The global survey by Gruber-Dorninger, Müller Gruber-Dorninger, Müller [29] exemplifies this trend, reporting prevalence rates of 68.6% for FBs, 61.1% for ZEA, 58.0% for DON, 41.6% for AFB_1_, and numerous emerging mycotoxins within the same feed samples. Third, contamination patterns increasingly reflect the globalization of feed ingredient supply chains, whereby raw materials originating from different climatic regions are blended during feed manufacture, facilitating simultaneous exposure to *Aspergillus*-, *Fusarium*-, *Penicillium*-, and *Alternaria*-derived metabolites.

Collectively, these findings demonstrate that differences among studies should not be interpreted as contradictory evidence but rather as the consequence of diverse environmental conditions, feed formulations, analytical capabilities, and sampling strategies. From a practical perspective, the widespread occurrence of multi-mycotoxin contamination suggests that routine monitoring programmes should move beyond the analysis of single toxins and adopt comprehensive multi-analyte methods capable of detecting both regulated and emerging mycotoxins. Likewise, mitigation strategies should be tailored to regional climatic conditions, ingredient sourcing, and feed management practices, rather than relying on universal approaches, to effectively reduce mycotoxin exposure in aquaculture systems.

## 5. Toxic Effects of Mycotoxins in Fish: Species-Specific Responses, Dose–Effect Relationships, and Comparative Insights

The compiled dataset demonstrates that mycotoxins exert a wide spectrum of toxic effects in fish, ranging from subclinical growth depression to severe organ damage, immunosuppression, reproductive impairment, genotoxicity, and mortality (Table 2, Table 3, Table 4, Table 5, Table 6, Table 7, Table 8 and Table 9). These effects are highly dependent on mycotoxin type, dose, exposure duration, and fish species, highlighting significant interspecies variability in susceptibility and toxicodynamics. Overall, the toxic effects of mycotoxins in fish are complex, species-dependent, and strongly influenced by exposure conditions. Collectively, the available evidence underscores the need for species-specific risk assessments and multi-mycotoxin management strategies in aquaculture. Importantly, the frequent co-occurrence of mycotoxins in feed suggests that real-world toxicity may be underestimated when single-toxin effects are considered in isolation.

The toxicological effects of mycotoxins in fish have become increasingly important with the expansion of aquaculture and the growing use of plant-based feed ingredients prone to fungal contamination. Research conducted over the past two decades demonstrates that mycotoxins exert a broad spectrum of adverse effects in fish, ranging from reduced growth performance and immunosuppression to severe organ damage, reproductive dysfunction, genotoxicity, carcinogenesis, and mortality. These toxic responses vary considerably depending on the mycotoxin type, dose, duration of exposure, environmental conditions, and fish species, highlighting significant interspecies differences in susceptibility and toxicodynamics.

AFB_1_ primarily targets the liver because of its central role in xenobiotic metabolism, leading to hepatotoxic, immunotoxic, mutagenic, and carcinogenic effects. Tilapia species (*Oreochromis* spp.) are among the most sensitive freshwater fish studied. Even low dietary AF concentrations of 25–50 µg/kg significantly reduce weight gain, feed efficiency, and survival in tetrahybrid red tilapia [118], while higher concentrations exceeding 100 µg/kg cause severe growth depression and mortality [94,95]. Histopathological investigations consistently report hepatocyte disorganization, cytoplasmic vacuolization, necrosis, fatty liver, and inflammatory infiltration following chronic exposure [102,112]. Hematological disturbances, including reduced hemoglobin, erythrocyte counts, and serum proteins, together with elevated liver enzymes such as ALT and AST, further confirm systemic toxicity [96,98]. At extreme concentrations of approximately 3000 µg/kg, AFB_1_ induces DNA damage, metabolic disruption, and impairs digestive function [93]. However, some studies suggest that very low exposure levels may not immediately induce tumor-like lesions, indicating possible threshold effects under short-term exposure conditions [61].

Carp species exhibit moderate sensitivity to AFs compared with tilapia. In common carp (*Cyprinus carpio*), dietary AFB_1_ exposure reduces growth performance and feed conversion efficiency while inhibiting digestive enzyme activity and promoting toxin accumulation in the hepatopancreas and gonads [115]. Higher doses induce tissue necrosis and immune cell infiltration [117]. In contrast, gibel carp (*Carassius auratus gibelio*) demonstrate relatively higher tolerance at low exposure levels, with fewer pathological alterations reported [37]. Catfish species, particularly channel catfish (*Ictalurus punctatus*), are highly susceptible to AFB_1_ toxicity, exhibiting severe hematological disturbances, tissue necrosis, and multi-organ damage at concentrations ranging from 2.2 to 12 mg/kg [137]. African catfish (*Clarias* spp.) exposed to combined AFB_1_ and FB contamination develop anemia, leukopenia, and marked growth reduction [146]. Similarly, rohu (*Labeo rohita*) experience mortality, immunosuppression, and poor growth following AFB_1_ exposure [101,121].

Marine fish species also exhibit pronounced sensitivity to AFs. Sea bass (*Dicentrarchus labrax*) exposed to low AFB_1_ concentrations develop behavioral abnormalities, hemorrhages, and severe systemic pathology [66]. Sturgeon (*Huso huso*) display neurological dysfunction, liver cancer, and severe physiological impairment after relatively short exposure periods [136]. Chronic AF exposure in rainbow trout has long been associated with liver tumor formation and enlargement of internal organs, making this species a classical model for AF carcinogenicity studies [61]. Overall, tilapia and catfish appear particularly vulnerable to AF-induced growth suppression and hepatotoxicity, whereas marine species often exhibit severe systemic and behavioral responses even at lower concentrations.

**Table 3:** Toxic effects of fumonisins related in different species of fish.

Mycotoxins	Fish/Species	Dose (µg/kg)	Administration/Duration	Toxic Effects	References
FBs	Tilapia (*Oreochromis* *niloticus*) fingerlings	20,000, 40,000 and 60,000	Ration—4 weeks	Reduction in messenger RNA levels, reduced expression of the CASP7 gene	[147]
FB_1_	Tilapia (*Oreochromis niloticus*) juvenile	200, 300, 500, 600, 800 and 1000	Ration—6 weeks	0.60 mg/kg (LD50)	[148]
FBs	Channel catfish (*Ictalurus* *punctatus*)	5000–15,000	Feed—oral (6 weeks)	Reduction in weight gain; hematocrit, erytrocytes, hemoglobin and protein constituents decreased	[149]
FB_1_	Channel catfish (*Ictalurus* *punctatus*) fry	20,000–40,000	10 weeks	Reduced weight gain and feed intake. Increase in feed conversion ratio. Reduction in hematocrit. Increased ratio of free sphinganine/free sphingosine in liver.	[150]
FB_1_	Channel catfish (*Ictalurus punctatus*)	10,000	Feed: oral (10 weeks)	Decreased leukocyte count, increased haematopoietic activity of blood-forming tissues	[138]
FB_1_	Channel catfish (*Ictalurus* *punctatus*)	80,000 320,000 or 720,000	Feed: oral (14 weeks)	Reductions in growth, lower hematocrit and red cell counts, and higher white cell counts	[151]
FB_1_	Channel catfish (*Ictalurus* *punctatus*)	20,000, 80,000, 320,000 or 720,000	Feed: oral (14 weeks)	Swollen hepatocytes in the liver with lipid-containing vacuoles, lymphocyte infiltration, and scattered necrotic hepatocytes	[151]
FB_1_	Channel catfish (*Ictalurus punctatus*)	300, 20,000, 80,000, 320,000 and 720,000	Ration—14 weeks	320 mg/kg (LD70)	[151]
FB_1_	Channel catfish (*Ictalurus punctatus*)	300–720,000	10–14 weeks	Reduction in weight gain and feed intake. Lower hematocrit and red cell counts. Pale liver and kidneys. Liver lesions, white foci of subcapsular adipocyte hyperplasia, foci of swollen and shrunken hepatocytes and of hepatocellular necrosis. Yellow-white ventral portion of the liver. Higher mortality due to Cytophaga columnaris infection.	[151]
FB_1_	Channel catfish (*Ictalurus punctatus*)	35,000–313,000	5 weeks	No general negative health effects were noted. Mild enteritis.	[152]
FB_1_	Nile tilapia (*Oreochromis niloticus*)	40,000, 70,000, 150,000	Feed: oral (8 weeks)	Lower mean weight gains	[153]
FB_1_	Nile tilapia (*Oreochromis niloticus*)	150,000	Feed: oral (8 weeks)	Haematocrit was decreased and ratio between free sphinganine and free sphingosine (SA/SO) in the liver increased	[153]
FBs	Nile tilapia (*Oreochromis niloticus*)	60,000	-	Decreased levels of messenger RNA, reduced expression of the CASP7 gene	[147]
FBs	Gibel carp (*Carassius* *auratus gibelio*)	20, and 2000	Feed: oral (24 weeks)	Gonadosomatic index (GSI), absolute brood amount (AF), relative brood amount (RF), and oocyte diameter were significantly lower	[128]
FB_1_	Common carp (*Cyprinus carpio*)	10,000 and 100,000	Feed: oral (6 weeks)	Blood vessels, liver, exocrine and endocrine pancreas, excretory and haematopoietic kidney, and heart and brain were sensitive	[154]
FB_1_	Common carp (*Cyprinus carpio*)	500 and 5000	Feed: oral (6 weeks)	Loss of body weight and alterations of haematological and biochemical parameters in target organs	[155]
FB_1_	Common carp (*Cyprinus carpio*)	5000	Feed: oral (6 weeks)	Increase in bacterial infection	[155]
FBs	Rohu (*Labeo rohita*)	2500 and 5000	Intraperitoneal (10 weeks)	Reduction in production of oxygen radicals by neutrophils	[111]
FBs	Rohu (*Labeo rohita*)	1250, 2500 and 5000	Intraperitoneal (10 weeks)	Reduction in total protein and globulin levels	[111]
FBs	Rohu (*Labeo rohita*)	10,000, 20,000 and 40,000	Feed: oral (8 weeks)	Total erythrocyte count, total leucocyte count, hemoglobin count, and nitroblue tetrazolium decreased	[101]
FBs	Sea bass (*Dicentrarchus* *labrax* L.)	180	Feed: oral (4 days)	Loss of equilibrium, rapid opercular movement, and hemorrhages of the dorsal skin surface	[66]
FBs	Rainbow trout (*Oncorhynchus mykiss*) juvenile	1190	Feed: oral (3 weeks)	Mortality	[156]
FB_1_	Rainbow trout (*Oncorhynchus mykiss*)	23,000	Feed: oral (42 weeks)	Cancer promoter	[157]
FB_1_	Catfish (*Clarias* spp.)	7500	-	Decreased total red blood cell count, hematocrit, hemoglobin, and serum enzymes	[158]
FB_1_	Catfish (*Clarias* spp.)	80,000	-	Reduction body length, weight gain, specific growth rate, and feed conversion. Increases in serum cholesterol, triglycerides, and the sphinganine-sphingosine ratio	[159]

Abbreviations: ALB = albumin; ALP = alkaline phosphatase; ALT = alanine transaminase; AST = aspartate aminotransferase; FCR = feed conversion ratio; FE = feed efficiency; FI = feed intake; HIS = hepatosomatic index; LYZ = lysozyme; Nrf2 = NF-E2-related factor 2; SGR = specific growth rate; TJ = tight junction; TP = total protein; WG = weight gain.

Fumonisins, constitute another important class of mycotoxins affecting fish. Table 3 above present reported toxicity of fuminisins to diverse species of fish. Their toxicity is primarily associated with disruption of sphingolipid metabolism, leading to cellular dysfunction and apoptosis. In tilapia, FB exposure causes reduced growth performance and altered expression of apoptosis-related genes such as CASP7 [147]. Channel catfish exhibit dose-dependent reductions in growth, hematological parameters, and liver function when exposed to fumonisin-contaminated diets, with adverse effects reported at approximately 20–80 mg FB_1_/kg feed, while an estimated LD_70_ of about 320 mg/kg body weight has been reported under acute exposure conditions [151]. Common carp demonstrate multi-organ sensitivity involving the liver, pancreas, kidney, and brain following dietary exposure to 10–100 mg FB_1_/kg feed [154], while rohu show immunosuppression and decreased serum protein concentrations after exposure to approximately 20–60 mg FB_1_/kg feed [111]. Compared with AFs, FBs generally require higher doses to induce acute toxicity; however, chronic exposure (typically ≥4–12 weeks in experimental feeding trials), can still produce significant physiological and immunological impairment, including reduced growth, altered liver enzyme activity, oxidative stress, and suppression of immune responses.

**Table 4:** Toxic effects of deoxynivalenols related in different species of fish.

Mycotoxins	Fish/Species	Dose (µg/kg)	Administration/Duration	Toxic Effects	References
DON	Common carp (*Cyprinus carpio* L.) juvenile	352; 619 and 953	Ration—4 weeks	Cytotoxic effects on immune cells	[160,161]
DON	Common carp (*Cyprinus carpio* L.) juvenile	352; 619 and 953 L	Ration—6 weeks	Increased lipid peroxidation in the liver, kidney and spleen	[160,161]
DON	Atlantic salmon (*Salmo* *salar* L.)	3700	Feed: oral (8 weeks)	Reduction in feed intake and decrease in specific growth rate	[162]
DON	Atlantic salmon (*Salmo* *salar* L.)	3700	Feed—oral (15 weeks)	Reduced weight gain	[163]
DON	Atlantic salmon (*Salmo salar*)	55,000		Impairs the epithelial barrier and modulates cytokine signaling	[164]
DON	Rainbow trout (*Oncorhynchus mykiss*)	300, 1000, 1500, and 2000	Ration—12 weeks	Reduction in weight gain and growth, low feed conversion	[165]
DON	Rainbow trout (*Oncorhynchus mykiss*) fingerlings	4500 and 10,500	Ration—8 weeks	Reduction in weight gain, low feed conversion, altered proteolytic enzyme activity, elevated messenger RNA levels and nutrient retention	[166]
DON	Rainbow trout (*Oncorhynchus* *mykiss*) juvenile	300, 800, 1400, 2000, 2600	Ration—8 weeks	Reduction in weight gain, low growth rate and feed conversion, morphological changes in the liver	[165]
DON	Rainbow trout (*Oncorhynchus mykiss*)	2600	Feed: oral (8 weeks)	Decrease in growth, feed intake, feed efficiency, and protein and energy utilization.	[167]
DON	Rainbow trout (*Oncorhynchus mykiss*)	300–2600	Feed—oral (56 days)	Reduced weight gain	[167]
DON	Channel catfish (*Ictalurus punctatus*)	20,000	Feed—oral (7 weeks)	Reduced feed conversion ratio	[168]
DON	Channel catfish (*Ictalurus punctatus*)	5000–10,000	Feed: oral (8 weeks)	Mortality	[168]
DON	Rainbow trout (*Oncorhynchus mykiss*)	1964	23 days	Gastrointestinal and liver hemorrhaging. Probable anaemia, lower values of erythrocyte haemoglobin, corpuscular haemoglobin concentration and haemoglobin in red blood. Probable decrease in the metabolism’s intensity, decrease in glucose, cholesterol and ammonia. Lesions in the caudal kidney, severe hyaline droplet.	[169]
DON	Rainbow trout (*Oncorhynchus mykiss*)	300–2600	8 weeks	Reduction in weight gain and growth rate. Decrease in feed intake and feed efficiency. Decrease in recovered energy, energy retention efficiency, retained nitrogen and nitrogen retention efficiency. Lower whole-body crude protein concentration. Lesions in the liver, subcapsular hemorrhage and edema, fatty infiltration and altered hepatocytes.	[170]
DON	Rainbow trout (*Oncorhynchus mykiss*)	3300 and 6400	4 weeks	Reduction in feed intake and weight gain. Increase in respiratory burst. Reduction in susceptibility to *Flavobacterium psychrophilum* infection.	[171]
DON	Rainbow trout (*Salmo gairdneri*)	1–13	4 weeks	Reduction in feed intake and feed conversion efficiency. Reduction in weight.	[167]
DON	Grass carp (*Ctenopharyngodon idella*)	1515	-	Poor body formation, histopathological lesions, oxidative damage, decreased antioxidant capacity, and cell apoptosis	[172]
DON	Grass carp (*Ctenopharyngodon idella*)	2100	-	Decreases in weight gain, thermal-unit growth coefficient, feed intake, and feed efficiency. Increases in dead cells in the pyloric caeca and decreases in mitotic cells in the pyloric caeca	[173]

DON is particularly associated with reduced feed intake, impaired nutrient utilization, and immunotoxicity. Salmonids, especially Atlantic salmon (*Salmo salar*) and rainbow trout (*Oncorhynchus mykiss*), are highly sensitive to DON contamination as presented in Table 4. Dietary exposure reduces feed consumption, growth performance, immune responsiveness, and vaccine efficacy in Atlantic salmon at dietary concentrations of approximately 0.5–6 mg/kg feed, with more pronounced effects on feed intake and growth reported at 4–6 mg/kg [162]. In rainbow trout, dietary DON concentrations of 0.3–2.6 mg/kg feed administered for eight weeks resulted in dose-dependent reductions in feed intake and growth performance, with morphological changes in the liver observed at 2.6 mg/kg [167,169]. Freshwater species also exhibit adverse responses at comparatively lower concentrations. In common carp, exposure to 0.352, 0.619, and 0.953 mg/kg feed for six weeks altered erythrocyte characteristics and antioxidant responses, particularly at the higher concentration, although growth performance was not affected, similarly, juvenile grass carp fed diets containing 0.027–1.515 mg/kg feed for 60 days showed oxidative damage, apoptosis, intestinal barrier disruption, and impaired growth, with several adverse intestinal responses occurring from approximately 0.318 mg/kg, while more pronounced growth impairment was observed at concentrations ≥ 0.636 mg/kg [160,172]. These findings indicate that the sensitivity to DON varies considerably among fish species and that both relatively low concentrations and higher dietary levels can adversely affect physiological, metabolic, immune, and growth-related endpoints. Salmonids, particularly Atlantic salmon and rainbow trout, appear comparatively sensitive to DON-induced reductions in feed intake and growth, highlighting DON as an important concern in aquaculture systems.

**Table 5:** Toxic effects of zearalenones related in different species of fish.

Mycotoxins	Fish/Species	Dose (µg/kg)	Administration/Duration	Toxic Effects	References
ZEA	Zebrafish (*Danio rerio*)	1000 ng·L^−1^	Feed: oral (26 weeks)	Affect growth and changed relative fecundity from one generation to another	[174]
ZEA	Zebrafish (*Danio rerio*) embryos	100–1000	Water—oral (5 days)	Edemas, a developmental phenotype reminiscent to the heart and soul mutant and the lack of the pigmentation gap at the base of the caudal fin	[175]
ZEA	Zebrafish (*Danio rerio*)	1	140 days	Increased weight gains and body length on female fish. Feminization, shift towards female sex. Induction of plasma vitellogenin in females.	[174]
ZEA	Zebrafish (*Danio rerio*)	100–3200	42 days	Reduced of the relative spawning frequency. Reduced relative fecundity. Induction of plasma vitellogenin in male fish.	[176]
ZEA	Zebrafish (*Danio rerio*)	1000 and 3200 ng·L^−1^	Feed: oral (6 weeks)	Reduced spawning frequency	[176]
ZEA	Rainbow trout (*Oncorhynchus mykiss*)	2000	96 weeks	Increased feeding efficiency and growth rate. Modulation of the adaptative and innate immune system. Inflammation likely caused by pathogen infection. Changes in kidney morphology leading to atypical kidney structure and fibrosis, reddish spots displaying disorganized kidney morphology with inflammatory areas and granulomatous structures, whiteish spots or translucent, whiteish nodules. Rupture blood cells. Kidney inflammation was suggested to be due to Tetracapsuloides bryosalmonae infection. Increased albumin to globulin ratio (although not statistically significant). Decreased in lymphocytes concentration.	[177]
ZEA	Rainbow trout (*Oncorhynchus mykiss*) juvenile	1810	Feed: oral (10 weeks)	No effects on growth and may accelerate sexual maturation of female fish	[178]
ZEA	Common carp (*Cyprinus carpio* L.)	332, 621 and 797	Feed: oral (4 weeks)	No effect on growth but effects on haematological parameters	[179]
ZEA	Common carp (*Cyprinus carpio* L.) juvenile	332; 621 and 797	Ration—4 weeks	Cytotoxic effects on immune cells	[179]
ZEA	Grass carp (*Ctenopharyngodon idella*)	797	-	Influence on white blood cell counts, genotoxicity by micronucleus formation in erythrocytes	[179]
ZEA	Black tiger shrimp (*Penaeus monodon* *Fabricius*)	500,000 and 1,000,000	Feed: oral (10 weeks)	Histological changes in hepatopancreatic tissue	[180]

ZEA differs from other major mycotoxins because its primary effects involve endocrine disruption and reproductive dysfunction (Table 5). Zebrafish (*Danio rerio*) are especially sensitive and have become important experimental models for studying ZEA toxicity. In a life-cycle study, exposure to 320 and 1000 ng/L ZEA altered sex ratio toward females from 320 ng/L, whereas increased female body condition and vitellogenin induction were observed at 1000 ng/L [174]. During embryonic development, ZEA produced concentration-dependent toxicity, with developmental abnormalities reported from 250 µg/L, more pronounced cardiac, ocular, and body-axis abnormalities at ≥500 µg/L, and reported LC10 and LC50 values of 335 and 893 µg/L, respectively [175]. In rainbow trout, dietary exposure of 2 mg/kg feed affected immune-related parameters following long-term feeding while juvenile common carp fed 0.332, 0.621, or 0.797 mg/kg ZEA for four weeks, alters immune responses, causes kidney inflammation, and disrupts hematological parameters [177,179].

**Table 6:** Toxic effects of ochratoxin A related in different species of fish.

Mycotoxins	Fish/Species	Dose (µg/kg)	Administration/Duration	Toxic Effects	References
OTA	Shrimp (*Aristaeomorpha* spp.)	2000, 4000, and 8000	Ration—8 weeks	Reduction in weight gain and growth, low feed conversion	[181]
OTA	Channel catfish (*Ictalurus punctatus*) juvenile	500, 1000, 2000, 4000 and 8000	Ration—8 weeks	Reduction in weight gain, low feed conversion, Haematocrit reduction, mortality	[182]
OTA	Channel catfish (*Ictalurus punctatus*) juvenile	500–8000	8 weeks	Reduction in body weight gain. Decrease in feed conversion rate. Reduction in hematocrit. Lesions in the liver and posterior kidney, increased incidence and severity of melanomacrophage centers in the hepatopancreatic tissue and posterior kidney and reduced number or absence of exocrine pancreatic cells surrounding the portal veins. Increased mortality.	[182]
OTA	Sea bass (*Dicentrarchus labrax*)	50, 100, 150, 200, 250, 300, 350, and 400	Ration—4 days	0.3 mg/kg (LD50)	[66]
OTA	Sea bass (*Dicentrarchus labrax* L.)	500–4000	24–96 h	Behavioral changes, sluggish movement, loss of equilibrium, rapid operculum movement, changes in the swimming pattern and respiratory manifestation. Muscular seizures prior to death. Hemorrhagic patches on the dorsal surface. Erosion of the fins and rusty spot formation in the belly region and dorsal musculature. General congestion of the kidney and gills. Spots of congestion on the periphery of the liver. Increased mortality.	[66]
OTA	Marine Sea Bass (*D. labrax* L.)	290	Feed—oral	Reduction in the rate of survival by up to 50%; congestion of the kidneys and gills	[66]
OTA	Nile tilapia (*Oreochromis niloticus*)	3% of fish body weight	14 days	Reduce weight gain, feed intake, final weight and feed conversion rate. Sluggish swimming. Lesions in the liver, kidneys and spleen. Enlargement and congestion of kidney and liver. Dilation of blood vessels and necrosis of the kidney, degeneration and necrosis of hepatocytes. Pericarditis and myocarditis. Increased levels of alanine aminotransferase, aspartate transaminase, creatine and urea. Congested gills. Enlarged gallbladder. Reduction in total protein, albumin and globulin. Neutropenia. Higher mortality.	[183]
OTA	Grass carp (*Ctenopharyngodon idella*)	2406	-	Reduction in weight gain, growth, feed efficiency, and specific growth rate. Oxidative damage and increased intestinal permeability. Destruction of intestinal apical junctional complexes	[184]
OTA	Atlantic salmon (*Salmo salar*)	200–2400	8 weeks	Increase in alkaline phosphatase, cholesterol, total protein, albumin and aspartate transaminase. Increased mRNA expression of immune marker in the spleen.	[185]
OTA	Catfish (*Clarias* spp.)	1000	-	Biochemical disturbances and oxidative stress in the liver and kidney, histological changes, and increased DNA fragmentation in the kidney	[186]
OTA	Tilapia (*Oreochromis niloticus*) fingerlings	80, 160	Intraperitoneal —8 weeks	0.08 mg/kg (LD50)	[187]
OTA	Common carp (*Cyprinus carpio*) juvenile	4000	Feed: oral (6 weeks)	Mortality (80.49%)	[188]
OTA	Black tiger shrimp (*Penaeus monodon*)	1000	Feed: oral (8 weeks)	No negative impact in shrimp	[189]

OTA has been associated with adverse effects in several fish species, although responses vary with species, dose, and exposure duration. Table 6 presents some reported toxicity effects. In channel catfish, dietary OTA exposure for 8 weeks reduced growth and caused hepatopancreatic lesions [182]. Nile tilapia fed a diet containing 0.5 mg/kg OTA for 10 weeks showed reduced survival (53%), impaired growth, hepatorenal lesions, and increased serum ALT, AST, urea, and creatinine concentrations [183]. In marine-reared sea bass, acute dietary exposure resulted in behavioral abnormalities, with a 96 h dietary LC50 of 9.23 mg/kg diet and a corresponding LC50 of 277 µg/kg body weight [190]. In Atlantic salmon, dietary OTA concentrations of 0.2–2.4 mg/kg feed administered for up to 8 weeks produced relatively small and inconsistent changes in selected clinical biochemical and immune-related parameters, with no significant effects on growth performance or liver histopathology [185].

**Table 7:** Toxic effects of T-2 related in different species of fish.

Mycotoxins	Fish/Species	Dose (µg/kg)	Administration/Duration	Toxic Effects	References
T-2	Rainbow trout (*Oncorhynchus mykiss*)	18,000	-	Decrease in corpuscular hemoglobin, plasma hemoglobin, corpuscular volume and nonspecific humoral immunity. Increased in erythrocyte counts and leukocyte and lymphocyte count	[191]
T-2	Tilapia (*Oreochromis niloticus*)	23,300	-	Mortality, reduction in weight gain and hepatosomatic index, damage to liver cells and myofibers	[192]
T-2	Rainbow trout (*Oncorhynchus* *mykiss*)	1000 and 1800	Ration—4 weeks	Oxidative stress, increased lipid peroxidation, Increased ceruloplasmin activity	[191]
T-2	Common carp (*Cyprinus carpio* L.)	5300		Decreased weight gain and feed efficiency, anemia, lymphopenia, oxidative stress, and changes in immune responses	[193]
T-2	Rainbow trout (*Oncorhynchus mykiss*)	10,000	Intraperitoneal (168 h)	Interfere with important biological processes (i.e., blood coagulation or iron-storage) and different cellular components (i.e., cytoskeleton)	[194]
T-2	Zebrafish (*Danio rerio*) embryos	0.20–0.80	Feed—oral (24 h of exposure)	Abnormalities in tail formation; cardiovascular defects; mortality upper 50%	[195]
	Zebrafish (*Danio rerio*)	3.247	Water—oral (4 days)	Estrogenic potency	[175]
T-2	Common carp (*Cyprinus carpio*) juvenile	1200, 2400, 4800 and 12,200	Feed: oral (3 weeks)	Decrease in growth and survival rate	[102]
T-2	Common carp (*Cyprinus carpio*) juvenile	1000 or 2000	Feed: oral (6 weeks)	Mortality	[188]
T-2	Common carp (*Cyprinus carpio*)	10,000 and 100,000	42 days	Reduction in body weight. Lesions in the liver, pancreas, kidney, heart, gallbladder and brain and damages in the blood vessels. Erythrodematitis lesions.	[154]
T-2	Common carp (*Cyprinus carpio*)	5300	Ration—4 weeks	Reduction in weight gain, low feed conversion, anemia, lymphopenia, oxidative stress	[193]
T-2	Zebrafish (*Danio rerio*) embryo	50, 100, 200, 250, 300, 400 and 800	Ration—24 h	0.40 (LD80) and 0.80 mmol/L (LD100)	[195]
T-2	Pacific whiteshrimp (*Litopenaeus vannamei*)	2400 and 4800	Feed: oral (3 weeks)	Antioxidant enzymes, superoxide dismutase (SOD) and glutathione peroxidase (GPx), total antioxidant capacity (TAOC), and also glutathione (GSH) content increased	[102]
T-2	Channel catfish (*Ictalurus punctatus*) juvenile	1250, 2500, and 5000	Feed: oral (8 weeks)	Reductions in growth and hematocrit values were adversely affected	[182]

Among trichothecenes, T-2 toxin is considered especially potent because of its strong cytotoxic and oxidative effects (Table 7). In rainbow trout, dietary exposure to T-2 toxin at 1.0 and 1.8 mg/kg feed for 28 days induced oxidative stress and altered antioxidant defenses, while increased lipid peroxidation was observed at the higher concentration of 1.8 mg/kg [191]. In common carp, dietary exposure to 5.3 mg/kg feed for four weeks caused hematological alterations, oxidative stress, increased lipid peroxidation in the liver and caudal kidney, and changes in immune responses, although no mortality occurred during the trial [193]. Zebrafish embryos are particularly sensitive to T-2 toxin, with exposure to 0.20 µmol/L or higher resulting in increased mortality and developmental malformations, including tail and cardiovascular defects, together with oxidative stress and apoptosis [195].

**Table 8:** Toxic effects of emerging mycotoxins related in different species of fish.

Mycotoxin	Fish/Species	Dose (µg/kg)	Administration/Duration	Toxic Effect	Reference
STC	Nile tilapia (*Oreochromis niloticus*)	Intragastric (4 weeks)	Intragastric (4 weeks)	Reduced body weight. Increase frequency of micronucleated red blood cells and chromosomal aberrations mainly in the kidney. Increased mortality. Accumulation in the fish’s musculature.	[196]
		1.6	4 weeks	Behavioral changes, unbalanced swimming. Darker color. Histopathological lesions in different organs hyperplastic proliferation of bronchial epithelium, necrobiotic changes in hepatic and splenic tissues and destruction of components of the spleen. DNA changes polymorphism band patterns.	[197]
STC	Rainbow trout (*Oncorhynchus mykiss*) embryos (gairdneri)	500	14 days	Increased incidence of hepatocellular carcinomas among survivors 1 year later.	[198]
STC	Rainbow trout (*Oncorhynchus mykiss*)	1.64 g·kg^−1^ bwt	Corn oil: oral (4 weeks)	Genotoxic and toxicopathological effects	[197]
STC	Rainbow trout (*Oncorhynchus mykiss*)	1190	Feed—oral (21 days)	Mortality	[156]
STC	Sea bass (*Dicentrarchus labrax* L.)	180	Feed—oral (96 h)	Loss of equilibrium, rapid opercular movement, hemorrhages of the dorsal skin surface.	[66]
MON	Channel catfish (*Ictalurus punctatus*)	20,000, 40,000, 60,000 and 120,000	Feed: oral (10 weeks)	Reductions in growth	[150]
MON	Channel catfish (*Ictalurus punctatus*)	60,000	Feed: oral (10 weeks)	Low hematocrit level and high serum pyruvate level	[150]
MON	Nile tilapia (*Oreochromis* *niloticus*) fingerlings	10,000–150,000	8 weeks	Reduction in weight gain. Higher feed conversion rates. Lower hematocrit. Increase ratio between free sphinganine/free sphingosine.	[153]

Emerging mycotoxins such as STC and MON have also attracted increasing attention (Table 8). STC, a biosynthetic precursor in the aflatoxin pathway, has been associated with genotoxic and carcinogenic effects in fish. In Nile tilapia, exposure to approximately 0.5 mg STC/kg body weight produced genotoxic effects, while exposure of rainbow trout embryos to STC resulted in hepatocarcinogenic responses [196,198]. MON also affects fish health; dietary exposure to approximately 1–8 mg MON/kg feed in young channel catfish resulted in impaired growth and alterations in hematological parameters, with responses varying according to dose and whether MON was administered alone or in combination with FB_1_ [150].

**Table 9:** Toxic effects of co-occuring mycotoxins in different species of fish.

Mycotoxin	Fish/Species	Dose (µg/kg)	Administration/Duration	Toxic Effect	Reference
AFB_1_ & FB_1_	African catfish (*Clarias gariepinus*) juvenile	2.0; 7.3; 17.6; 48.0; 93.0 L g/kg and 3000, 15,000, 24,500, 43,000, 83,000	Ration—8 weeks	Reduction in weight gain and length, low feed conversion	[146]
AFB_1_ & FB_1_	African catfish (*Clarias gariepinus*)	93 and 83	-	Anemia, leukopenia, reduced growth and weight gain. Decreased red blood cell count, hemoglobin concentration, and hematocrit	[146]
AFB_1_ & ZEA	Gold fish (*Carassius* spp.)	100 and 1000	-	Reduction in weight gain and specific growth	[142]
AFB_1_ & ZEA	Rainbow trout (*Oncorhynchus mykiss*)	25≥ 50≤	-	Decreased LYZ, TP, and ALB and increased inflammatory cytokines	[141]
AFB_1_ & ZEA	Gold fish (*Carassius* spp.)	100 and 1000	-	Reduction in weight gain and specific growth	[142]
AFB_1_ & ZEA	Red drum (*Sciaenops ocellatus*)	100–5000	7 weeks	Reduced weight gain and feed efficiency. Reduction in the whole-body lipid levels. Liver lesions. Reduced survival.	[100]
AFB_1_ & ZEA	Red drum (*Sciaenops ocellatus*)	250–100,000	8 weeks	Reduced weight gain. Reduced hematocrit. Histopathological changes in the liver, excess lipofuscin, irregularly sized hepatocellular nuclei and necrosis. Increased mortality.	[33]
AFB_1_ & ZEA	Gibel carp (*Carassius auratus* *Gibelio*)	3.2–991.5	12–16 weeks	No significant health effects were determined. Low residues were found in muscles (<2.32 g/kg).	[37]
AFB_1_ & ZEA	Gibel carp (*Carassius auratus* *Gibelio*) juvenile	3.2–28.6	3 months	Slight damages to the hepatopancreas. Low levels of toxin in the muscle (<4.08 g/kg).	[128]
DON & OTA	Atlantic salmon (*Salmo salar*)	6000 and 24, 000		Mortality, reduction in feed intake, feed efficiency, gain, length, and condition factor. Increased mRNA expression of immune markers in the spleen	[185]
DON & ZEA	*Tilapia* (*Oreochromis* *niloticus* and *Oreochromis* *mossambicus*) fingerlings	70, 310, 500, 920, 1150 and 100, 90, 210, 370, 980	Ration—8 weeks	Reduction in weight gain and growth, low feed conversion	[199]

The combined exposure of fish to multiple mycotoxins generally produced more severe adverse effects than single toxins, with growth impairment being the most consistently reported outcome (Table 9). Overall, co-contamination with AFs, FBs, ZEA, DON, and OTA adversely affected growth, feed utilization, hematological status, liver integrity, immune function, and survival in several fish species. The severity of toxicity varied with toxin combination, dose, exposure duration, and fish species, while low-dose AFB_1_–ZEA exposure in gibel carp produced comparatively minor effects.

Overall, the evidence demonstrates that mycotoxin toxicity in fish is highly species-specific and multifactorial. AFB_1_ remains the most toxic and widespread contaminant, primarily targeting liver function and growth, whereas DON predominantly affects feed intake and immunity in salmonids, ZEA disrupts reproductive and endocrine systems, and OTA and FBs induce consistent multi-organ toxicity. The frequent co-occurrence of multiple mycotoxins in aquafeeds suggests that real-world toxicity may be substantially underestimated when individual toxins are assessed in isolation, emphasizing the need for integrated multi-mycotoxin risk assessment and management strategies in aquaculture systems.

A comparison between farm-raised and wild-caught fish was added to clarify the likely source of mycotoxin contamination. The evidence summarized in this review is largely derived from controlled feeding studies in aquaculture species, indicating that contaminated feed is a major route of exposure in farm-raised fish. These studies consistently report dose-dependent reductions in growth, feed efficiency, hematological parameters, liver function, immune competence, and survival following dietary exposure to AFs, FBs, and DON. For example, Nile tilapia exposed to dietary AFB_1_ showed reduced growth, liver damage, hematological alterations, and mortality, while channel catfish fed FB-contaminated diets exhibited reduced weight gain, anemia, liver lesions, and increased susceptibility to secondary infections. In contrast, wild-caught fish are not routinely exposed to compounded aquafeeds; therefore, contamination detected in dried or processed wild fish is more likely related to fungal growth during drying, smoking, storage, transport, or marketing rather than feed-to-tissue transfer. The generally low muscle residues reported in some exposure studies further support limited carryover to edible tissues under many conditions. This comparison helps distinguish aquaculture-associated contamination from contamination introduced during post-harvest handling and storage. Examples supporting the farm-raised category include Nile tilapia exposed to AFB_1_ with reduced growth and liver injury, channel catfish exposed to FBs with anemia and liver lesions, Atlantic salmon exposed to DON with reduced feed intake and growth, and rainbow trout exposed to AFB_1_ with severe liver pathology and tumor-like lesions. Low muscle residues reported in gibel carp and other species suggest limited transfer to edible tissue under many exposure conditions. These findings support the distinction between feed-related contamination in aquaculture and post-harvest contamination in wild-caught fish.

## 6. Mycotoxin Regulation in Aquafeeds

The regulation of mycotoxins in aquafeeds remains fragmented worldwide, and aquaculture-specific regulatory limits are uncommon. In many jurisdictions, mycotoxin control in aquafeeds is addressed indirectly through general regulations or guidance applicable to animal feeds, rather than through limits established specifically for farmed fish. Consequently, risk management in aquaculture continues to rely largely on regulatory frameworks originally developed for poultry, swine, cattle, and other terrestrial animals, despite important physiological and nutritional differences between these production systems [1,25]. Such an approach provides a regulatory basis for controlling mycotoxin exposure but may not fully account for differences among fish species in feed intake, metabolism, toxin sensitivity, life stage, and the potential transfer of mycotoxins or their metabolites into edible tissues. In the European Union (EU), Commission Recommendation 2006/576/EC provides reference values for the presence of certain mycotoxins in products intended for animal feeding and remains one of the principal regulatory guidance frameworks relevant to aquafeeds [200,201]. These recommendations establish guidance values for AFB_1_, DON, ZEA, FBs, OTA, and T-2/HT-2 toxins in feed materials and complete feeds. Importantly, these values are guidance values for animal feed and are not legally binding maximum limits established specifically for fish or aquaculture feeds. For example, complete feeds intended for fish and other non-ruminant animals should not exceed 10 µg/kg AFB_1_, while guidance values of 5000 µg/kg DON and 10,000 µg/kg fumonisins have been established for complete and complementary feeds [200,201]. These values are intended to support risk management by limiting animal exposure to mycotoxins and reducing potential adverse effects on animal health and productivity.

For aquaculture, risk management should therefore extend beyond simply applying terrestrial-animal guidance values. A practical risk-management approach includes: (i) prevention and reduction in contamination in feed ingredients through good agricultural and storage practices; (ii) routine monitoring and testing of high-risk ingredients and complete feeds; (iii) identification of mycotoxin co-occurrence and cumulative exposure; (iv) application of appropriate mitigation measures where contamination is detected; (v) evaluation of species-, life-stage-, and dose-specific susceptibility; and (vi) assessment of potential carryover of mycotoxins or their metabolites into edible fish tissues. These measures are particularly important because aquafeeds frequently contain plant-derived ingredients that may introduce multiple mycotoxins simultaneously [1,25]. Accordingly, Table 10 should not be interpreted as a compilation of legally binding regulatory limits specifically established for fish feed. Rather, it summarizes selected EU guidance values for mycotoxins in animal feed that are applicable or potentially relevant to aquaculture in the absence of widely adopted fish-specific standards. The values should therefore be interpreted as reference points for risk assessment and feed management rather than as universally applicable maximum limits for aquafeeds.

However, the occurrence data summarized in this review demonstrate that mycotoxin contamination is predominantly reported from Africa, Asia, and the Middle East rather than Europe, highlighting a disparity between the geographical distribution of contamination and the availability of regulatory frameworks. Many African countries lack legally enforceable limits for mycotoxins in animal feeds, with regulatory efforts focusing primarily on human food commodities such as maize and peanuts [1,202]. Similarly, several countries in the Middle East and parts of Asia rely on general food safety legislation or voluntary quality standards without establishing specific maximum limits for aquafeeds. In contrast, countries such as China have developed national standards for selected mycotoxins in feed ingredients, whereas the United States regulates aflatoxins in animal feeds through action levels established by the U.S. Food and Drug Administration (FDA) but does not provide comprehensive regulations covering the wide range of mycotoxins now detected in commercial aquafeeds [1]. Consequently, considerable regional variation exists in regulatory requirements, surveillance capacity, and enforcement, making direct comparison of contamination data across countries difficult.

This regulatory heterogeneity partly explains the substantial differences in reported contamination frequencies among studies. Regions with well-established surveillance programmes and advanced analytical infrastructure, such as Europe, generally report comprehensive multi-mycotoxin datasets generated using LC-MS/MS, whereas many developing countries continue to monitor only AFs using ELISA or HPLC because of financial and technical constraints [27,29]. Therefore, apparent geographical differences in contamination may reflect differences in monitoring intensity and analytical capability as much as true environmental variation. Another important limitation of current regulations is that most guidance values were derived from toxicological studies conducted in terrestrial livestock rather than fish. Existing maximum limits are primarily based on adverse effects observed in poultry, pigs, cattle, and laboratory mammals, assuming exposure scenarios and toxicokinetic responses that may not accurately represent aquatic species [24,25]. Fish differ substantially from terrestrial animals in gastrointestinal physiology, nutrient metabolism, xenobiotic biotransformation, excretory pathways, and environmental exposure. Moreover, fish continuously interact with the aquatic environment, where additional stressors including water temperature, dissolved oxygen, stocking density, water quality, and infectious diseases may modify the toxicity of dietary mycotoxins [52]. Consequently, guidance values considered safe for terrestrial livestock may not provide equivalent protection for fish. Species-specific differences further complicate regulatory development. Numerous studies have demonstrated marked variation in mycotoxin sensitivity among fish species. Rainbow trout (*Oncorhynchus mykiss*) are highly susceptible to AFB_1_, developing hepatocellular carcinoma following chronic exposure to relatively low dietary concentrations, whereas Nile tilapia (*Oreochromis niloticus*) and common carp (*Cyprinus carpio*) generally tolerate higher exposure levels before comparable pathological changes occur [24,25]. Similarly, differences in growth rate, feeding behaviour, digestive physiology, and hepatic detoxification capacity influence susceptibility to FBs, DON, OTA, and ZEA. These interspecific differences suggest that a single universal regulatory limit may not adequately protect all cultured fish species. An additional challenge is that existing regulations predominantly address individual mycotoxins despite increasing evidence that commercial aquafeeds frequently contain multiple regulated and emerging mycotoxins simultaneously. Large-scale surveys summarized in this review consistently demonstrate the co-occurrence of AFs, FBs, DON, ZEA OTA, ENs, BEA, MON, and other metabolites within the same feed samples [29,60]. Experimental studies indicate that these mixtures may produce additive or synergistic toxic effects even when individual mycotoxins remain below current guidance values [203].

Therefore, regulatory approaches based exclusively on single-compound thresholds may underestimate the actual toxicological risk encountered under commercial aquaculture conditions. Collectively, these observations indicate that current regulatory frameworks require substantial refinement to better reflect the realities of modern aquaculture. Future regulations should move beyond adapting terrestrial livestock standards and instead incorporate fish species-specific toxicity data, chronic dietary exposure studies, regional differences in feed ingredients and climatic conditions, and the toxicological consequences of multi-mycotoxin exposure. Harmonization of international surveillance programmes, wider adoption of validated multi-analyte analytical methods, and the development of science-based guidance values specifically for aquaculture would substantially improve feed safety, facilitate international trade, and enhance consumer protection.

## 7. Conclusions

The evidence synthesized in this review demonstrates that mycotoxin contamination of fish feed and processed seafood is a widespread and increasingly complex challenge with significant implications for aquaculture sustainability, fish health, food security, and public health. The growing reliance on plant-based feed ingredients has increased the risk of contamination by toxigenic fungi, resulting in the frequent occurrence of both regulated and emerging mycotoxins, often as multi-mycotoxin mixtures. Among these, AFB1 remains the most prevalent and hazardous contaminant, particularly in tropical and subtropical regions. The widespread co-occurrence of mycotoxins and the resulting additive or synergistic toxic effects highlight the need to move beyond single-toxin risk assessments.

The reviewed evidence also demonstrates considerable interspecies variability in susceptibility to mycotoxin exposure, with chronic contamination adversely affecting fish growth, immunity, reproduction, and overall productivity. The detection of mycotoxins in processed seafood products further emphasizes the potential for human dietary exposure, especially since many mycotoxins remain stable during conventional processing.

Overall, effective management of mycotoxin contamination requires an integrated approach encompassing improved agricultural and storage practices, enhanced feed quality control, rapid monitoring systems, and harmonized regulatory frameworks that address both regulated and emerging mycotoxins, as well as the realities of multi-mycotoxin exposure. Coordinated efforts among researchers, the aquaculture industry, regulatory agencies, and policymakers will be essential to strengthen feed safety, protect aquatic animal and human health, and support the long-term sustainability of global aquaculture.

### Future Research Directions

Despite the growing number of studies on mycotoxins in aquaculture, major knowledge gaps remain regarding the toxicological significance of multi-mycotoxin exposure in fish. The evidence compiled in this review indicates that studies investigating co-occurring mycotoxins are still very limited, with most experimental work focusing on single compounds. Future research should therefore move beyond single-toxin models and systematically evaluate feed-relevant mycotoxin mixtures, particularly combinations frequently reported in aquafeeds such as AFB_1_–FB_1_, AFB_1_–DON, AFB_1_–ZEA, DON–ZEA, and FB_1_–ZEA. These investigations should prioritize chronic low-dose exposure scenarios that reflect realistic field conditions rather than short-term high-dose experimental designs. Particular attention should be given to interactive effects, including additive, synergistic, and antagonistic responses on growth, immunity, oxidative stress, intestinal integrity, reproduction, and survival.

Based on the occurrence and toxicity data summarized in this review, several fish species warrant higher research priority. Nile tilapia (*Oreochromis niloticus*) should remain a major focus because it is one of the most widely farmed fish globally and has been used extensively in mycotoxin studies, allowing comparative assessments across toxins and exposure conditions. African catfish (*Clarias gariepinus*) deserves increased attention because the limited co-contamination studies available already suggest marked sensitivity to combined AFB_1_ and FB_1_ exposure, while the species is economically important throughout sub-Saharan Africa. Common carp (*Cyprinus carpio*) should also be prioritized owing to its global production importance and its frequent inclusion in feed studies. In addition, more information is needed for rainbow trout (*Oncorhynchus mykiss*), particularly regarding chronic multi-mycotoxin exposure under intensive production systems, as salmonids are known to be highly susceptible to several mycotoxins.

Future investigations should further integrate toxicokinetic and residue studies to clarify feed-to-tissue transfer, persistence, and potential human dietary exposure from contaminated fish products. Standardized experimental protocols, including harmonized diet formulations, exposure durations, analytical methods, and reporting units, are urgently needed to improve comparability among studies and enable quantitative risk assessment and meta-analysis.

Because mycotoxin contamination of aquafeeds is a global challenge but occurs under diverse agroecological and economic conditions, research priorities should also be tailored to regional realities. In many developing countries, limited laboratory infrastructure and regulatory resources constrain routine surveillance. Practical priorities should therefore include the development of low-cost rapid screening tools, region-specific occurrence databases for locally used feed ingredients, farmer-oriented feed storage interventions, and evaluation of affordable mitigation options such as locally available adsorbents, fermentation technologies, and improved post-harvest handling. Strengthening regional laboratory networks, proficiency testing programs, and capacity building in mycotoxin analysis and risk assessment would substantially improve surveillance and evidence-based regulation.

## 8. Methodology

This review was conducted as a systematic literature review with narrative synthesis following the Preferred Reporting Items for Systematic Reviews and Meta-Analyses (PRISMA 2020) guidelines [204]. The review was designed to synthesize current evidence across diverse aquaculture production systems, fish species, geographical regions, and feed ingredients while identifying emerging trends, knowledge gaps, and future research priorities. Particular emphasis was placed on studies investigating multi-mycotoxin exposure, naturally contaminated feeds, species-specific responses, and food safety implications associated with aquaculture products.

### 8.1. Search Strategy

A structured literature search was performed using multiple scientific databases, including Scopus, Web of Science, PubMed, ScienceDirect, and Google Scholar, supplemented by technical reports and publications from international organizations involved in food safety, feed safety, and aquaculture research. Searches were conducted to capture peer-reviewed journal articles, review papers, conference proceedings, book chapters, and relevant technical reports published in English.

The search strategy employed combinations of keywords and Boolean operators (AND and OR) to maximize the retrieval of relevant studies. Core search terms included “mycotoxins in fish feed,” “aquaculture and mycotoxins,” “mycotoxin contamination in aquafeed,” “toxic effects of mycotoxins in fish,” “co-occurrence of mycotoxins,” “fish health and mycotoxins,” “aflatoxins in aquaculture,” “fumonisins in fish feed,” “ochratoxin A in fish,” “zearalenone toxicity in fish,” and “emerging mycotoxins in aquaculture.” Additional searches incorporated specific fish species, aquaculture systems, feed ingredients, and geographical regions to improve coverage and contextual relevance.

### 8.2. Eligibility Criteria

Studies were considered eligible for inclusion if they:

- Reported the occurrence or concentration of one or more mycotoxins in aquaculture feeds or feed ingredients.
- Examined co-occurrence or multi-mycotoxin contamination patterns.
- Investigated toxicological, physiological, biochemical, immunological, reproductive, or histopathological effects of mycotoxins in fish.
- Evaluated exposure risks, species susceptibility, growth performance, feed utilization, or food safety implications.
- Provided original research data, surveillance findings, or experimentally validated evidence relevant to aquaculture production.

Studies were excluded if they:

- Focused exclusively on terrestrial livestock without aquaculture relevance;
- Did not provide sufficient methodological or analytical information;
- Were duplicate publications; or
- Were not available in English.

### 8.3. Study Selection and Data Extraction

Retrieved records were screened for relevance based on titles, abstracts, and full-text assessment. Eligible studies were selected according to their relevance to mycotoxin occurrence, contamination levels, co-contamination patterns, affected fish species, toxicological outcomes, and implications for aquaculture productivity and food safety.

Data extracted from each study included:

- Study location and geographical region;
- Fish species investigated;
- Type and concentration of mycotoxins detected;
- Feed ingredients and feed categories examined;
- Co-occurrence patterns of multiple mycotoxins;
- Experimental design and exposure conditions;
- Reported physiological, biochemical, immunological, reproductive, and histopathological effects.

### 8.4. Data Synthesis and Analysis

The selected literature was systematically organized into thematic categories based on mycotoxin type, fish species, geographical distribution, contamination levels, co-contamination profiles, toxicological effects, and mitigation approaches. A narrative synthesis approach was employed to integrate findings across studies and identify recurring patterns, regional differences, and emerging concerns. Unlike previous reviews that primarily focus on individual mycotoxins or specific fish species, this review adopts an integrated perspective by simultaneously examining global contamination trends, multi-mycotoxin interactions, species-specific susceptibility, and food safety consequences across aquaculture systems. This comprehensive approach enables the identification of critical knowledge gaps and research priorities necessary for improving feed safety, fish health, and sustainable aquaculture production worldwide.

## Figures and Tables

**Table 1:** Occurrence of mycotoxins in fish feed.

Region	Sample Size	Mycotoxin	Detection Techniques	% Contamination	Concentration (Range) (µg/kg)	References
Africa	45	AFB_1_	HPLC	-	1.2–3.7	[56]
		AFB_2_			0.3–2.3	
		AFG_1_			13–35.3	
		AFG_2_			2.4–7.8	
	81	AFB_1_	-	100	97–403.4	[58]
		FBs			0.1–4.06	
	34	AFs	ELISA	-	3.64	[59]
		AFB_1_			1.82	
	78	AFs	HPLC	97.44	14.7–93.6,	[60]
		AFB_1_			14.7–43.6	
		AFG_1_			<155.8	
		DON			40.4–819.9	
		DON-3-G			46.8–97.5	
		ZEA			38.0–757.9	
		αZEL			22.2–288.4	
		β–ZEL			16–79.8	
		FBs			63–2076.6	
		FB_1_			63–1427.4	
		FB_2_			68.9–649.2	
		ECO			37.6–64.3	
		ECR			<24.9	
		ENV			<21.9	
		ESN			38.4–144.2	
		ETA			29.3–1895.6	
		αECP			41–81.3	
		ERG			20.7–2055.3	
		FUSX			<56	
		HT-2			41.6–411.8	
		NEO			<177.7	
		NIV			40.3–76	
		AOH			36.2–43.3	
		AME			<94.5	
		ENA			<26.1	
		ENA_1_			13.5–23.8	
		ENB			38.8–150	
		ENB_1_			12.9–43.5	
		EN			19.4–186.7	
		CUL			42.3–288.7	
		BEA			15.9–841.8	
		STC			30.5–3517.1	
		MON			218.9–2583.4	
	81	AFs		83.95	0.07–39.7	[61]
	25	AFs		96	0.07–20	[40]
	52	AFs	LC-MS/MS	100	2.4–126	[62]
		FBs			33.2–2834.6	
		DON			69.1–755.4	
		NIV			45–732.5	
		AOH			2–91.3	
		T-2			<LOD	
		ADONs			22.5–63.2	
		DAS			0.7–1.1	
	120	AFBs	HPLC	100	1.1–49	[63]
	-	DON	ELISA	17	-	[64]
	50	AFs	-	40	LOD-150	[39]
	10	AFs, OTA, T-2	TLC	-	-	[65]
	-	AFB_1_	AflatestTM immunoaffinity	-	0.28–32	[66]
			column method			
	-	AFB_1_	-	50	-	[67,68]
		ZEA		8	-	
		DON		17	-	
		FBs		58	-	
		OTA		12	-	
Asia	21	AFs	ELISA	95.24	1.49–15.9	[69]
		OTA			1.49–7.63	
	414	AFs	LC and GC-MS/MS	2.42	0.32–28.3	[70]
		OTA			0.32–127.7	
	125	AFs	-	-	0.2–5.5	[71]
		T-2 + HT-2			1.7–18.5	
		OTA			0.1–2.1	
	12	AFs	LC-MS/MS	100	0.5–32	[72]
		DON			10–396	
		ZEA			10–153	
		15-ADON			10–139	
		FBs			10–993	
		OTA			0.5–3	
	86	AFB_1_	-	67.44	0.46–68.5	[32]
	15	DON	HPLC	66.67	0.25–2.5	[73]
		ZEA			0.2–5	
	-	AFs	-	-	LOD-67.35	[74]
		AFB_1_	-	15	-	[67,68]
		ZEA		63	-	
		DON		83	-	
		FBs		51	-	
		OTA		25	-	
		AFB_1_	-	88	-	
		ZEA		14	-	
		DON		22	-	
		FBs		56	-	
		OTA		49	-	
	-	AFB_1_	-	71	-	
		ZEA		37	-	
		DON		34	-	
		FBs		55	-	
		OTA		28	-	
	-	AFB_1_	-	37	-	
		ZEA		-	-	
		DON		11	-	
		FBs		67	-	
		OTA		50	-	
Europe	9	15-ADON	LC-MS/MS	100	21–55	[75]
		BEA			0.5–30	
		ENA_1_			0.47–1.9	
		ENB			1.1–21	
		ENB_1_			0.7–7.9	
		FB_1_			<18–136	
		FB_2_			<27–105	
	87	AFs	ELISA	100	1–17.5	[76]
		FBs			0.01–0.92	
	60	OTA	HPLC	-	0.01–0.28	[77]
	27	AFs	LC-MS-MS	-	0.4	[78]
		DON			30	
	10	ENs	LC-QTOF-MS	-	-	[79]
	6	AFs	MIP	100	32–106	[80]
	-	ENs	LCMS/MS-LIT	-	1.3–103	[81]
	2	ADONs	-	100	LOD-50	[82]
		AFs			1–2.5	
		BEA			06-May	
		FB_1_			LOD-100	
		HT-2			LOD-50	
		NIV			LOD-100	
		OTA			LOD-2	
		T-2			5–1816	
		ZEA			LOD-1	
		DON			LOD-50	
		DAS			LOD-5	
	5	FBs	LC-MS/MS	100	6.4–754	[83]
		DON			19.4–79.2	
		15-ADON			8.1–13.6	
		HT-2			LOD-5	
		T-2			LOD-0.1	
	20	ENA		100	0.6–3.4	[84]
		ENA_1_			0.3–6.5	
		ENB			0.1–3.2	
		ENB_1_			0.15–10	
		BEA			0.1–6.6	
	10	FB_1_		100	20–100	[85]
		ZEA			20–100	
	11	DON	LC-DAD and LC-FL	100	66–825	[35]
		ZEA			3–511	
	19	ENB	LC-MS-MS	-	1.26–44.65	[86]
	94	AFs	LC-MS/MS	100	1.1–418.7	[87]
		AFM_1_			1.2–17.7	
		DON			5.4–334	
		FBs			19.7–6097.9	
		NIV			5.5–65.2	
		ZEA			0.2–263	
	3	ZEA	LC-FL	-	10.3–81.8	[88]
	-	AFB_1_	-	ND	-	[67,68]
		ZEA		25	-	
		DON		71	-	
		FBs		ND	-	
		OTA		ND	-	
	-	AFB_1_	-	19	-	
		ZEA		41	-	
		DON		64	-	
		FBs		51	-	
		OTA		25	-	
	-	AFB_1_	-	33	-	
		ZEA		14	-	
		DON		36	-	
		FBs		56	-	
		OTA		41	-	
North America	-	AFB_1_	-	21	-	[67,68]
		ZEA		14	-	
		DON		50	-	
		FBs		27	-	
		OTA		21	-	
South America	15	AFB_1_	HPLC-FLD	93.33	0.1–16.5	[89]
		OTA			2.2–31.6	
		ZEA			22.9–322.2	
	36	AFB_1_	ELISA	16.7	1.6–9.8	[44]
	28	AFs	-	100	<1.7–8.91	[26]
		FBs			187–222	
		DON			150–230	
		OTA			5–8.79	
		T-2			LOD-70.08	
		ZEA			20.4–159.76	
	60	AFB_1_	ELISA and TLC	90	<LOD	[41]
		FB_1_			0.3–4.94	
		OTA			<LOD	
	30	AF		-	<LOD	[46]
		FB			LOD-2587	
	-	AFB_1_	-	13	-	[67,68]
		ZEA		28	-	
		DON		21	-	
		FBs		76	-	
		OTA		16	-	
Global	226	AFB_1_	LC-MS/MS	41.6	-	[29]
		FBs		68.6		
		DON		58		
		ZEA		61.1		
		OTA		33.6		
		ENB		46.9		
		ENB_1_		54		
		BEA		35		
		Alternariol		40.7		
-	12	AFs	-	100	<LD	[25]
		DON			25.1–43	
		ZEA			<LD	
		OTA			1.2–501.7	
		ENB_2_			12.7–18.5	
		BEA			<12.7	
		FBs			<50	
		T-2 + HT-2			<25	
-	30	AFB_1_	HPLC	23.33	1.8–29.4	[90]
		AFB_2_			1–15.1	
		AFG_1_			1.3–6.6	
		OTA			3.5–10.7	
-	25	AFs	-	95.24	52–221	[91]
		DON			29–413	
		FBs			58–573	
		OTA			5–7	
		ZEA			53–233	
-	41	DON	-	100	20–282	[92]
		FBs			3420–7534	
		OTA			LOD-3	
		ZEA			6–306	

*n* = 4327 (2009/10) and *n* = 4200 (2012/2013). ND = Not detected; MIP = molecularly imprinted polymer; LCMS coupled to linear ion trap (LIT) mass spectrometer; LC-Q-TOF-MS = liquid chromatography coupled to hybrid quadrupole time-of-flight mass spectrometry. α-ZEL = alpha-zearalenol; β-ZEL = alpha-zearalenol; MON = Moniliformin; NIV = Nivalenol; DAS = diacetoxyscirpenol; ADONS = acetyldeoxynivalenols; AOH = Alternariol; CUL = culmorin; AME = alternariol monomethyl ether; FUSX = Fusarenon-X; ERG = ergosterol; α-ECP = alpha-ergocryptine; ESN = ergosinine; ECR = ergocristine; ETA = ergotamine.

**Table 2:** Toxic effects of aflatoxins related in different species of fish.

Mycotoxin	Fish/Species	Dose (µg/kg)	Administration/Duration	Toxic Effect	References
AFB_1_	Nile tilapia (*Oreochromis niloticus*)	3000	-	Decreased growth, feed efficiency and digestive enzymes, increased liver enzyme activities, and DNA damage	[93]
		200, 250	-	Mortality	[94]
		150	-	Mortality, reduction in weight gain and hepatosomatic index, damage to liver cells and myofibers	[95]
AFs		1.8–39.7	-	No tumor-like lesions were detected.	[61]
AFB_1_	Tilapia (*Oreochromis* *niloticus*) fingerlings	2000	Ration—14 weeks	Reduction in weight gain, low feed conversion; decreased haemoglobin concentrations in the blood, total erythrocytes, total proteins, albumin and globulins	[96]
	Tilapia (*Oreochromis* *niloticus*)	2000, 4000	-	Reduced weight gain and decreased body lipid content	[97]
		100	-	Increased liver enzymes, reduced growth rate, and weight gain	[20]
		200	Oral (10 weeks)	Decreased total erythrocyte and leucocyte count, serum liver enzymes leakage, hemoglobin count decreased; reduced weight gain and reduction in the rate survival rate	[98]
		150	-	Serious toxic impacts and negative effects on health performance	[99,100]
		10, 20, 40	-	Swollen hepatocytes, kidney mild hemorrhages, decrease total erythrocyte, and hemoglobin count	[101]
		793 and 1641	Oral (15 weeks)	Darkening of body surface	[102]
		793 and 1641	Oral (5 weeks)	Yellowing of the body surface	[102]
		2500	Oral (20 weeks)	Abnormal behavior	[102]
		245	Oral (20 weeks)	Feed efficiency rate decreased	[102]
		245, 638, 793, 1641	Oral (20 weeks)	Weight gain lowest	[102]
	Tilapia (*Oreochromis* *niloticus*, *O.* *aureus*) fingerlings	19–1641	Ration—20 weeks	Reduced weight gain and growth, yellowing skin, severe liver injury and decreased total protein and albumin content.	[102]
	Tilapia (*Oreochromis niloticus*)	3000	-	Lower SGR	[103]
	Nile tilapia (*Oreochromis niloticus*)	100	-	Severe liver tissue vacuolation and lipid accumulation	[104]
		100	-	Decreased growth	[105]
		100	-	Weight loss, changes in blood parameters, and liver necrosis	[106]
AFB_1_	Nile tilapia (*Oreochromis niloticus*)	5–38.62	Oral (10 weeks)	Survival rate reduced by up to 67%. Yellowing of the body surface	[107]
	Tetrahybrid red tilapia (*Oreochromis* spp.)	1250	-	Increased serum lysozyme activity, enhanced phagocytic ratio, and immunostimulatory effects	[108]
		<5	-	Pale gills, liver damage, poor growth rates, and immune suppression	[109]
		1250	-	Reduction in nonspecific immunity	[110,111]
		7500, 1125	-	Acute toxicity, Subchronic toxicity	[110,111]
	Tilapia (*Oreochromis niloticus*) fingerlings	250, 2500, 10,000, 100,000	Ration—8 weeks	Affect the hematocrit and growth performance. 100 mg/kg (LD60). Lipofuscin and irregularly hepatocellular nuclei, weight loss, severe hepatic necrosis, and mortality	[33]
	Nile tilapia (*Oreochromis* *niloticus*)	900–3000	25 days	Reduced growth feed intake. Histological changes in the liver neoplastic changes and fatty liver. Congestion of the kidney, shrinking of the glomeruli and melanosis. Decreased FI and growth rate, liver atrophy	[112]
		100	Oral (10 weeks)	Reduced the growth	[113]
		200	Oral (10 weeks)	Mortality (16.7%)	[113]
AFB_1_	Common carp (*Cyprinus carpio*)	500, 1000, 2000	-	Decreased weight gain, histopathological changes	[114]
AFB_1_	Common carp (*Cyprinus carpio*) Juvenile	50	Ration—8 weeks	Reduced growth and low feed conversion	[115]
AFs	Common carp (*Cyprinus carpio*) Juvenile	500, 700, 1400	Ration—3 weeks	Decrease in lysozyme activity and reduction of immunoglobulin in blood plasma	[116]
AFB_1_	Common carp (*Cyprinus carpio*) Juvenile	50	-	Reduced growth and increased feed conversion. Decreased protease, amylase, and lipase activities in the hepatopancreas and intestine. Accumulation in the hepatopancreas and gonads	[115]
	Common carp (*Cyprinus carpio*) Juvenile	400	-	Damage to bowel tissues, including necrosis, immune cell infiltration, and fibroplasia	[117]
AFB_1_	Rohu (*Labeo rohita*)	25, 50	-	Reduced growth performance, and growth depression	[118,119]
		100	-	Reduced growth indices and survival rate	[118,119]
		100	-	Cytoplasmic vacuolization in hepatocytes and hepatic tissue showing loss of membrane integrity, along with diffused hepatocytes and hyperplasia	[120]
AFB_1_	Rohu (*Labeo rohita*)	150	-	Mortality and reduced weight gain	[121]
AFB_1_	Rohu (*Labeo rohita*)	-	-	Decreased liver glycogen and total serum protein. Increased the blood level of glucose and urea	[122]
AFB_1_	Rohu (*Labeo rohita*)	10, 20 and 40	Feed—oral (8 weeks)	Total erythrocyte count, total leucocyte count, hemoglobin count and nitroblue tetrazolium decreased	[101]
AFB_1_	Rohu (*Labeo rohita*) fingerlings	7500, 11,250 and 13,750	Intraperotenial —10 days	13.75 mg/kg (LD60)	[110,111]
AFB_1_	Grass carp (*Ctenopharyngodon idella*)	85.94	-	Decreased expression of genes β-defensin-1, LEAP-2A, Mucin2, and LEAP-2B	[123]
AFB_1_	Grass carp (*Ctenpharyngodon idella*)	100	-	Reduction in specific growth rates. Reduction in red blood cells, hematocrit, hemoglobin concentration, corpuscular volume, white blood cell, and lymphocyte count. Increase in AST, ALT, glucose, urea and creatinine. Elevation of total serum proteins and albumin level. High feed conversion rate	[124]
AFB_1_	Grass carp (*Ctenopharyngodon idella*)	147	-	Poor growth performance and deformities	[125]
AFs	African catfish (*Clarias gariepinus*)	46.5; 1.4; 58.9, and 2.1	-	Decrease in serum protein and albumin. Increase in aspartate aminotransferase, alanine aminotransferase, and serum creatinine	[63]
AFB_1_	Silver catfish (*Rhamdia quelen*) juvenile	1177	Ration—3 weeks	Affects neurotransmitters; hyperlocomotion; increased Acetylcholinesterase activity; reduced sodium potassium pump activity	[126]
AFB_1_	Silver catfish (*Rhamdia quelen*)	1177	Ration—3 weeks	Changes in the cerebral phosphotransferase network	
AFB_1_	Striped catfish (*Pangasius* *hypophthalmus*) fingerlings	50; 100; 250; 500 and 1000 lg/kg	Ration—8 weeks	Reduction in weight gain, increase in hepatosomatic rate, increase in adipose somatic index, elevated levels of alanine aminotransferase and aspartate aminotransferase	[72]
AFB_1_	Striped catfish (*Pangasius* *hypophthalmus*)	1000	-	Reduction in weight gain, increase in the hepatosomatic and somatic adipose index. Elevated levels of alanine aminotransferase and aspartate aminotransferase. Low disease resistance	[72]
AFB_1_	Catla (*Catla catla*)	50	-	Mortality, reduction in weight gain, growth and specific growth rate, and histological changes in the liver	[127]
AFB_1_	Gold fish (*Carassius* spp.)	1646.5	-	Changes in gonadosomatic index, absolute brood amount, relative brood amount, and oocyte diameter	[128]
AFB_1_	Rainbow trout (*Oncorhynchus mykiss*)	5000	-	Decreases in hepatosomatic index, fat content, serum total protein, albumin, and globulin levels	[129]
AFB_1_	Turbot (*Scophthalmus maximus* L.) juvenile	100	8 weeks	Negatively affected liver catalase activity and intestinal microbiota	[130]
AFB_1_	Yellow catfish (*Pelteobagrus fulvidraco*)	234	-	Increased the total lipid and triglycerides contents in muscle, while decreased flesh contents of moisture, total protein, and phospholipids, and reduced myofiber diameter	[131]
AFB_1_	Lambari fish (*Astyanax altiparanae*)	10	-	Accumulation in fish liver and muscle	[132]
AFB_1_	Yellow catfish (*Pelteobagrus fulvidraco*)	1000	-	Mortality, reduction in weight gain, final body weight, and specific growth rate, and an increase in feed conversion ratio	[133]
AFB_1_	White sturgeon (*Huso huso*) (*A. ruthenus* ♂ × *A. baeri* ♀) juvenile	80 40	-	High mortality, decreased haematocrit value, nuclear hypertrophy, and hyperchromasia	[134]
AFB_1_	White sturgeon (*H. huso*)	75 100	-	Altered feed conversion and weight gain and decreases in growth	[135]
AFB_1_	White sturgeon (*Huso huso*) juvenile	10	<15 days	Decreased feed intake and weight loss. Increased weakness in the performance. Changes in swimming behavior. Hemorrhagic skin lesions in the head and abdomen. Yellow spots in the pectoral area. Curvature of the spinal cord. Accumulation of exudes liquid in the ventricular and kidney. Hepatitis and liver cancer. Hyperinflammation of gallbladder.	[136]
	Sea bass (*Dicentrarchus* *labrax* L.)	18	42 days	Abnormal behaviour, sluggish movements, swimming imbalance, rapid opercular movement and loss of equilibrium. Muscular seizures prior to death. Hemorrhages and yellow patches in the dorsal skin surface. Ascites. Hemorrhagic fluid in the abdominal cavity. Darkening of the body surface. Internal generalized congestion and pale discoloration of liver, kidney and gills. Severe distention of the gallbladder. Changes in eye opacity and exophthalmia. Increase in serum transaminases and alkaline phosphatase activities. Decrease in plasma proteins, albumin and globulin. Emaciation. Residual high levels (0.005 mg/kg) in the musculature.	[66]
AFB_1_	Channel catfish (*Ictalurus punctatus*)	12,000	10 days	Regurgitation of the stomach contents. Discoloration of the gills, livers, kidneys, spleen, stomach and intestines. Reduction in hematocrits, hemoglobin concentrations and erythrocyte counts. Histological lesions in the intestinal mucosa. Necrosis of hematopoietic tissues, hepatocytes, pancreatic acinar cells and gastric glands. Reduction in the volume of red pulp and the number of leukocytes in the spleen. Dilation of the renal tubular lumens.	[137]
AFB_1_	Channel catfish (*Ictalurus punctatus*)	2200–10,000	10 weeks	Reduced growth rate. Reduced hematocrit, hemoglobulin concentration and erythrocyte count. Increase in leukocyte count. Apparent hepatocellular necrosis and necrosis of the gastric glands. Dilation and changes in the profile of the head kidney. Increased hematopoietic activity of blood-forming tissues. Accumulation of iron pigments in the intestinal mucosal epithelium.	[138]
AFB_1_	Mosquitofish (*Gambusia affinis*)	4640	-	Acute toxicities and mortality	[139]
AFB_1_	Rainbow trout (*Oncorhynchus mykiss*)	50	-	Destruction of intestinal villi	[140]
AFB_1_ & ZEA	Rainbow trout (*Oncorhynchus mykiss*)	25≥ 50≤	-	Decreased LYZ, TP, and ALB and increased inflammatory cytokines	[141]
AFB_1_ & ZEA	Gold fish (*Carassius* spp.)	100 and 1000	-	Reduction in weight gain and specific growth	[142]
AFB_1_ & ZEA	Red drum (*Sciaenops ocellatus*)	100–5000	7 weeks	Reduced weight gain and feed efficiency. Reduction in the whole-body lipid levels. Liver lesions. Reduced survival.	[100]
AFB_1_ & ZEA	Red drum (*Sciaenops ocellatus*)	250–100,000	8 weeks	Reduced weight gain. Reduced hematocrit. Histopathological changes in the liver, excess lipofuscin, irregularly sized hepatocellular nuclei and necrosis. Increased mortality.	[33]
	Gibel carp (*Carassius auratus* *Gibelio*)	3.2–991.5	12–16 weeks	No significant health effects were determined. Low residues were found in muscles (<2.32 g/kg).	[37]
AFB_1_ & ZEA	Gibel carp (*Carassius auratus* *Gibelio*) juvenile	3.2–28.6	3 months	Slight damages to the hepatopancreas. Low levels of toxin in the muscle (<4.08 g/kg).	[128]
AFs	Rainbow trout (*Oncorhynchus mykiss*)	1.8–39.7	-	Enlarged liver with histological changes, white or yellow nodules or cyst swellings, tumor-like lesions, irregular cords of abnormal hepatocytes, necrosis and hemorrhages. Enlarged hearts and kidneys. Ascites. Swollen abdomen.	[61]
AFB_1_	Tambaqui (*Colossoma macropomum*) fingerlings	500	-	Decreases in the WG, FI, and FE	[143]
AFB_1_	Stellate sturgeon *Acipenser stellatus*	1500, 1850, 2300, 2850, 3500	-	Increased liver enzymes (AST, ALT, and ALP) and mortality rate	[144]
AFs	Stellate sturgeon (*Acipenser stellatus*)	1500 3500	-	8% mortality 50% mortality	[145]

Abbreviations: ALB = albumin; ALP = alkaline phosphatase; ALT = alanine transaminase; AST = aspartate aminotransferase; FCR = feed conversion ratio; FE = feed efficiency; FI = feed intake; HIS = hepatosomatic index; LYZ = lysozyme; Nrf2 = NF-E2-related factor 2; SGR = specific growth rate; TJ = tight junction; TP = total protein; WG = weight gain.

**Table 10:** EU guidance values for selected mycotoxins in animal feeds relevant to aquaculture.

Mycotoxin	Feed/Material Category	EU Guidance Value (µg/kg)	Relevance to Aquaculture
AFB_1_	Feed materials	20	Reference value; not fish-specific
AFB_1_	Complete feeds for non-ruminant/non-pig/non-poultry animals	10	Relevant to fish feeds
AFB_1_	Complementary feeds for non-ruminant/non-pig/non-poultry animals	20	Relevant to aquaculture
DON	Cereals/cereal products, except maize by-products	8000	Ingredient reference
DON	Maize by-products	12,000	Ingredient reference
DON	Complete and complementary feeds	5000	Relevant to aquafeeds
ZEA	Cereals/cereal products, except maize by-products	2000	Ingredient reference
ZEA	Maize by-products	3000	Ingredient reference
OTA	Cereals/cereal products	250	Ingredient reference
FBs	Maize and maize products	60,000	Ingredient reference
FBs	Complete and complementary feeds	10,000	Relevant to aquafeeds
T-2/HT-2	Cereals/cereal products, except oat bran	500	Ingredient reference

## Data Availability

No new data were created or analyzed in this study. Data sharing is not applicable to this article.

## Supplementary Materials

The following supporting information can be downloaded at: https://www.mdpi.com/article/10.3390/toxins18090383/s1. Table S1: Reports of frequently isolated fungi from fish feeds [205].
