# Supplementary material for: Global Occurrence, Co-Contamination Patterns, and Toxic Effects of Mycotoxins in Fish Feed: A Multi-Species Perspective Review
Source: Toxins (Basel). 2026 Sep 6;18(9):383. doi: 10.3390/toxins18090383 (PMC13611123; doi:10.3390/toxins18090383)
Supplement: Supplementary file 1 [file toxins-18-00383-s001.zip › toxins-4462741-supplementary.pdf]

Review

Supplementary Materials: Global Occurrence, Co-Contamination Patterns, and Toxic Effects of Mycotoxins in Fish Feed: A Multi-Species Perspective Review

Queenta Ngum Nji and Patrick Berka Njobeh

Table S1: Reports of frequently isolated fungi from fish feeds

Feed Source	Sample size	Country	Fungal isolates	Frequency of isolation (%)	References
Fish feedstuff	30	Egypt	*Aspergillus* spp. (*A. niger, A. flavus, A. fumigatus, A. versicolor, A. terreus)*	86.66 (43.33, 30, 6.66. 3.33, 3.33)	[90]
			Penicillium sp.	23.33	
			Fusarium sp.	10	
			Mucor sp.	6.66	
			Rhizopus sp.	3.33	
Tilapia feeds	25	Egypt	*A. flavus*	48	[40]
			A. niger	40	
			A. fumigatus	8	
			A. ochraceus	4	
			Penicillium sp.,	40	
			Cladosporium sp.	8	
			Candida sp.	40	
Fish feeds and ingredients	52	East Africa	*A. flavus*	55	[62]
			A. tamarii	9	
			Mucor sp.	9	
			Phoma sp.	6	
			A. niger	6	
			E.rubrum	3	
			P. chrysogenum	3	
Commercial and formulated fish feeds	121	Kenya	*Asperigillus* sp.	51	[205]
			Mucor sp.*	56	
			Rhizopus sp.	50	
			Saprolegnia sp.	43	
			Penicillium sp.	31	
Fish feeds and ingredients	54	Brazil	*Penicillium* sp.	83	[44]
			Aspergillus sp.	67	
			Rhizopus sp.	23	
			Cladosporium sp.	20	
Rainbow trout feed	28	Argentina	*Cladosporium cladosporioides*	54	[26]
			Eurotium repens	21	
			Eurotium rubrum	14	
			Mucor sp.	7	
			A. versicolor	4	
			P. crustosum	4	
			P. expansum	4	

			P. chrysogenum	4	
Fish feeds	86	Iran	*A. flavus*	47	[32]
			A. parasiticus	16	
			A. niger	14	
			A. ochraceus	10	
			A. fumigates	9	
			A. versicolor	5	
			A. carbonarius	5	
			A. nomius	5	
			A.ustus	5	
			Fusarium sp.	26	
			Eurotium sp.	10	
			Penicillium sp.	42	
			Mucor sp.	10	
			Cladosporium sp.	5	
			Alternaria sp.	5	
Fish feed	60	Brazil	*Cladosporium sp.*	85	[41]
			P. citrinum	71	
			A. flavus	35	
			A. niger	36	
			Eurotium sp.	20	
			Wallemia	40	
			Aureobasidium	10	
			Mucor	10	
			Nigrospora sp.	10	
Fish feed	36	Brazil	*A. flavus*	61	[42]
			A. fumigates	7	
			A. terreus	2	
			A. candidus	2	
			A. oryzae	7	
			A. penicillioides	2	
			Eurotium sp.	19	
			Penicillium sp.	19	
			Cladosporium sp.	16	
			Fusarium sp.	8	
Tilapia feed	30	Mexico	*Aspergillus flavus*	7	[46]
			Fusarium sp.	7	

Fish feed	15	Baghdad	*Penicillium sp.*	36.4	[73]
			Fusarium sp.	24.5	
			Aspergillus sp.	20	
			Mucor sp.	11.1	
			Alternaria sp.	8	
Sea bass feed	87	Portugal	*Aspergillus niger*	39	[45]
			Aspergillus glaucus	30	
			Penicillium sp.	29	
			Cladosporium sp.	29	
			Fusarium sp.	25	
Rainbow trout feed	-	Iran	*A. flavus*	61	[74]
			A. niger	20	
			A. ochraceus	1	
			A. fumigates	2	
			A. clavatus	1	
			Penicillium sp.	14	
			Absidia sp.	12	
			Mucor sp.	2	
			Alternaria sp.	2	
Fish feed	50	Egypt	*A. flavus*	56	[39]
			A. niger	4	
			A. ochraceus	2	
			A. candidus	8	
			Penicillium sp.	22	
			Mucor sp.	50	
			Cladosporium sp.	2	
			Rhizopus sp.	4	
Fish feed	10	Egypt	*A. flavus, A. clavatus, A. ochraceous, A. parasiticus, A. sydowii, A. terreus, A. versicolor, P. chrysogenum and Trichoderma viride.*	-	[65]
